# A subpopulation of high IL-21-producing CD4^+^ T cells in Peyer’s Patches is induced by the microbiota and regulates germinal centers

**DOI:** 10.1038/srep30784

**Published:** 2016-08-08

**Authors:** Leigh Jones, Wen Qi Ho, Sze Ying, Lakshmi Ramakrishna, Kandhadayar G. Srinivasan, Marina Yurieva, Wan Pei Ng, Sharrada Subramaniam, Nur H. Hamadee, Sabrina Joseph, Jayashree Dolpady, Koji Atarashi, Kenya Honda, Francesca Zolezzi, Michael Poidinger, Juan J. Lafaille, Maria A. Curotto de Lafaille

**Affiliations:** 1 Singapore Immunology Network (SIgN), Agency for Science, Technology and Research (A*STAR), Singapore; 2 Department of Microbiology, Yong Loo Lin School of Medicine, National University of Singapore, Singapore; 3 School of Biological Sciences, Nanyang Technological University, Singapore; 4 Skirball Institute and Department of Pathology, New York University School of Medicine, New York, NY, USA; 5 Department of Microbiology and Immunology, Keio University School of Medicine, Tokyo, and RIKEN Center for Integrative Medical Sciences (IMS), Yokohama, Japan; 6 Division of Pulmonary, Critical Care and Sleep Medicine, Department of Medicine and Department of Cell Biology, New York University School of Medicine, New York, NY, USA.

## Abstract

The production of IL-21 by T follicular helper (Tfh) cells is vital in driving the germinal centre reaction and high affinity antibody formation. However, the degree of Tfh cell heterogeneity and function is not fully understood. We used a novel IL-21eGFP reporter mouse strain to analyze the diversity and role of Tfh cells. Through the analysis of GFP expression in lymphoid organs of IL-21eGFP mice, we identified a subpopulation of GFP^+^, high IL-21 producing Tfh cells present only in Peyer’s Patches. GFP^+^Tfh cells were found to be polyclonal and related to GFP^−^Tfh cells of Peyer’s Patches in TCR repertoire composition and overall gene expression. Studies on the mechanisms of induction of GFP^+^Tfh cells demonstrated that they required the intestinal microbiota and a diverse repertoire of CD4^+^ T cells and B cells. Importantly, ablation of GFP^+^ cells resulted in a reduced frequency of Peyer’s Patches IgG1 and germinal center B cells in addition to small but significant shifts in gut microbiome composition. Our work highlights the diversity among IL-21 producing CD4^+^ Tfh cells, and the interrelationship between the intestinal bacteria and Tfh cell responses in the gut.

T follicular helper (Tfh) cells are crucial to the development of T cell-dependent antibody responses^1^,^2^. These activated CD4^+^ T helper cells establish cognate interactions with B cells within lymphoid follicles and germinal centers (GC) to mediate affinity maturation and differentiation of memory B cells and plasma cells. Tfh cells are identified by high expression of CXCR5, CD40L, inducible T cell costimulator (ICOS) and programmed cell death protein1 (PD1)^3^,^4^,^5^,^6^. Tfh cell differentiation requires reciprocal interactions of activated T helper cells with B cells, made possible by downregulation of CCR7 expression, upregulation of CXCR5, and localization at the T-B borders in secondary lymphoid organs^6^. High expression of the master transcription factor Bcl6 induced by T-B cell interaction drives the Tfh differentiation program^4^,^7^,^8^

Tfh cells characteristically produce the cytokine IL-21, and differ from Th1, Th2 and Th17 cells^9^,^10^, although they may also produce IL-4, IL-17 and IFNγ depending upon differentiation conditions^11^. IL-21 is essential for optimal B cell responses, supporting GC B cell proliferation and plasma cell differentiation while promoting class switching to IgG, and inhibiting class switching to IgE^12^,^13^,^14^. Accordingly, mice lacking IL-21 or IL-21R exhibit low levels of IgG1, IgG2b and IgG3, and high levels of IgE^12^,^15^. There is evidence that IL-21 is also important in the gut, where it potentiates IgA production induced by TGFβ and retinoic acid (RA)^13^,^16^. IgG is also induced in the gut, but its function has only recently began to be understood. IgG responses were shown to be important to eliminate virulent intestinal *Citrobacter rodentium*^17^ and to protect against systemic infections by enteric bacteria *E.coli* and *Salmonella*^18^. Under homeostatic conditions IL-21 is abundant in Peyer’s Patches (PP), where stimulation from the microbiota maintains constitutive GC and immune reactivity^19^,^20^,^21^.

The microbiota is essential for the formation of the organized immune system in the gut, and mutual feedback interactions between bacteria and the immune system maintain intestinal homeostasis^21^,^22^,^23^. An imbalance in the composition of the intestinal bacteria population, including reduced diversity, is associated with several inflammatory conditions^24^,^25^,^26^,^27^. It is thus important to understand the mechanisms whereby indigenous microbia in the gut induce homeostatic responses.

During development, intestinal bacteria colonization results in the differentiation of germinal centers in PP and intestinal lymphoid follicles. Germ-free mice exhibit under-developed gastrointestinal associated lymphoid tissue (GALT) and small PP^28^, while manipulating specific microbial populations in the gut using antibiotics can impact the induction of mucosal Th17 and Treg cells^29^,^30^. To date little is known about the induction, repertoire and functional heterogeneity of Tfh populations in the gut. In particular it is not known if different Tfh populations mediate production of IgA and IgG in the gut.

To study the induction and function of Tfh cells, we developed a novel IL-21eGFP BAC transgenic reporter mouse strain. Using the IL-21eGFP strain, we identified a subpopulation of highly-differentiated GFP^+^Tfh cells in murine PP that is induced by the microbiota and regulates GC and IgG1 B cell responses in PP.

Our results demonstrate heterogeneity among Tfh cells in the gut mucosal environment, and an interdependence between highly differentiated PP Tfh cells, B lymphocytes, and the indigenous intestinal bacteria. The new experimental system described here could facilitate the study of antigenic determinants, cellular interactions and environmental factors that induce highly differentiated intestinal Tfh cells, potentially advancing the understanding of homeostatic adaptive responses in the gut.

## Results

### GFP expression in IL-21eGFP reporter mice identifies a subpopulation of Tfh cells in Peyer’s Patches

**Figure 1:**
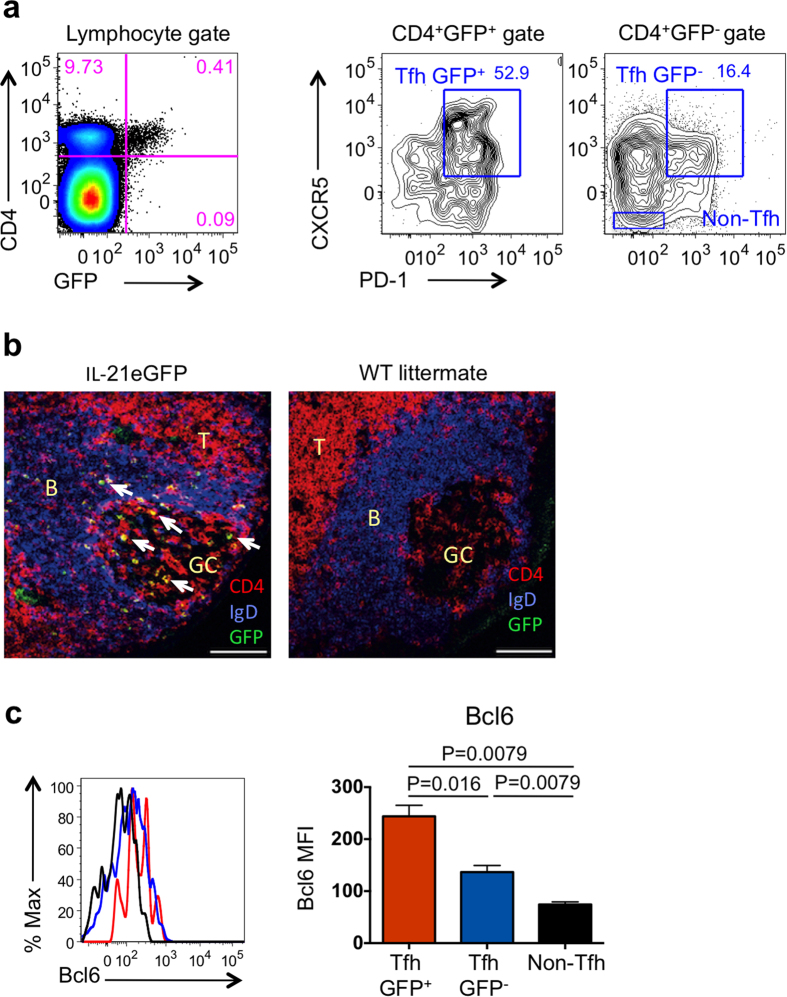
GFP expression defines a subpopulation of Tfh cells in PP of IL-21eGFP mice. (**a**) Flow cytometry analysis of PP cells from IL-21eGFP mice demonstrates GFP expression in CD4^+^ T cells (left plot). A higher percentage of CXCR5^+^PD-1^+^ Tfh cells is contained within GFP^+^ (middle plot) than GFP^−^ CD4^+^ cell (right plot) populations. (**b**) Identification of GFP^+^ (green), CD4^+^ (red) and IgD^+^ (blue) cells in frozen sections of PP from IL-21eGFP mice. Arrows indicate T cells co-expressing GFP and CD4 (yellow); scale bars = 100 μM; germinal centre (GC), B cell follicles (B) and T cell areas (T) are indicated. (**c**) Flow cytometry histograms show expression of Bcl6 in gated PP CD4^+^ T cell subsets from IL-21eGFP mice. The bar graph on the right shows mean ± s.e.m. of mean fluorescence intensity (MFI) values from 5 samples. Data are representative of two experiments. Statistical significance was determined by unpaired, two-tailed, Mann-Whitney U-test.

To study the heterogeneity of Tfh cells, we generated a BAC transgenic IL-21eGFP reporter mouse strain expressing a DTR-GFP fusion protein under the control of the IL-21 promoter and associated regulatory DNA sequences (Supplementary Fig. S1a). We identified a founder line displaying constitutive GFP expression in CD4^+^ T cells from PP (Fig. 1a and Supplementary Fig. S1b). GFP^+^ expressing cells were rare among CD4^+^ cells of mesenteric lymph nodes (MLN), were absent from other LN, spleen and thymus (Supplementary Fig. S1b), and were found at a very low frequency among intestinal lamina propria CD4^+^ T cells. No GFP expression was detected in NK, CD8^+^ or MHC II^+^ cells (data not shown). GFP was not induced in CD4^+^ T cells in spleen or peripheral LN of IL-21eGFP mice after intraperitoneal immunization with OVA in alum, or after subcutaneous immunization with KLH in alum (data not shown). Thus, in contrast to data obtained with other IL-21-reporter mice^31^,^32^,^33^, GFP expression in IL-21eGFP mice was restricted to a subpopulation of CD4^+^ T cells in PP.

Flow cytometry analysis demonstrated that over half of the GFP^+^ cells in PP expressed high levels of CXCR5 and PD1 (Fig. 1a), consistent with the Tfh phenotype. The remaining PP GFP^+^ cells expressed lower levels of CXCR5 or PD1. Approximately 10% of the total CXCR5^+^PD1^+^CD4^+^ Tfh population in PP of IL-21eGFP mice expressed GFP (Supplementary Fig. S1c). Immunofluorescence labeling of frozen sections from PP of IL-21eGFP mice showed that CD4^+^GFP^+^ cells were mainly found within GC and nearby B cell areas (Fig. 1b). Thus GFP expression appeared to mark a subpopulation of PP Tfh cells.

We then analyzed the expression of other genes that are specifically upregulated or downregulated in Tfh cells using flow cytometry and quantitative real time PCR (QPCR). Flow cytometry analysis of GFP^+^Tfh, GFP^−^Tfh cells, and non-Tfh CD4^+^ T cells from PP of IL-21eGFP mice showed that both Tfh populations expressed the Tfh master transcription factor Bcl6, but the expression was higher in GFP^+^Tfh than in GFP^−^Tfh cells (Fig. 1c). A similar expression pattern was observed for IL-21 and ICOS, although the differences between Tfh populations did not reach statistical significance (Supplementary Fig. S2a,b). GFP^+^Tfh also cells expressed significantly higher levels of TIGIT (the negative regulator T cell Ig and ITIM domain) than GFP^−^Tfh cells (Supplementary Fig. S2c). TIGIT was previously described to be expressed in Tfh cells^34^. CD226 and IL-7rα, known to be downregulated during differentiation of Tfh cells^10^,^34^, were expressed at lower levels in GFP^+^Tfh cells than in GFP^−^Tfh cells and non-Tfh cells (Supplementary Fig. S2c).

Global transcriptomic analysis comparing GFP^+^Tfh, GFP^−^Tfh and non-Tfh cells corroborated the identity of the two former populations as bona fide Tfh cells, and showed a high degree of similarity between these cells (Supplementary Fig. S3 and Supplementary Tables 1–3). *Bcl6*, *ICOS*, *Pdcd1*, *Maf*, *Tigit*, *CD44, Bach2*, *Bcl2* and *Smad7* were among the differentially expressed genes (DEGs) in GFP^+^Tfh and GFP^−^Tfh cells compared with non-Tfh cells (Supplementary Fig. S3a and Supplementary Table 1). We identified a subset of DEGs that showed differential expression between GFP^+^Tfh and GFP^−^Tfh cells (Supplementary Fig. S3b,c and Supplementary Tables 2 and 3). Importantly, the direction of change - dowregulation or upregulation - relative to the non-Tfh cells was the same for the GFP^+^Tfh cells and GFP^−^Tfh cells, but the change was more pronounced in the GFP^+^Tfh cells (Supplementary Fig. S3b,c and Supplementary Tables 2 and 3). Among the downregulated DEGs expressed at lower levels in GFP^+^Tfh than GFP^−^Tfh were *CCR7*, *Sell*, *Cmah*, *IL7r* and *Myc* (Supplementary Fig. S3b and Supplementary Table 2), and among the upregulated DEGs expressed at higher levels in GFP^+^Tfh than GFP^−^Tfh were *CXCR5* and *IL21* (Supplementary Fig. S3c, and Supplementary Table 3). The comparison between the PP Tfh DEGs identified in our studies and non-PP Tfh DEGs identified in two other mouse studies^35^,^36^ demonstrated significant overlap (Supplementary Table 4). Eighteen Tfh DEGs were identified in all three studies. Among these were signature Tfh genes, such as *Icos*, *Bcl6*, *Pdcd1*, *CXCR5* and *IL21*. Many DEGs were described in only one of the studies, possibly reflecting methodological differences as well as true variations between the Tfh populations analyzed.

In summary, we described a new IL-21eGFP reporter mouse strain in which GFP marks a PP-specific subpopulation of Tfh cells. GFP^+^Tfh cells exhibit higher expression of several signature Tfh genes than GFP^−^Tfh cells, and their overall gene expression suggests that they have a more differentiated Tfh phenotype.

### T cell receptor (TCR) repertoire analysis indicates that GFP^+^Tfh^+^ are closely related to GFP^−^Tfh^+^ in Peyer’s Patches

To study the clonal relatedness between GFP^+^Tfh and GFP^−^Tfh cells, we examined their TCR repertoires. If GFP^+^Tfh and GFP^−^Tfh cells were rapidly interchangeable in response to microenvironmental cues, their TCR repertoires would be highly overlapping, and different from that of non-Tfh cells. If GFP^+^ and GFP^−^Tfh cell populations were unrelated, there would be minimal overlap of the TCR repertoires. An intermediate situation would arise if GFP^+^Tfh cells originated from GFP^−^Tfh cells and constituted a fairly stable subpopulation.

**Figure 2:**
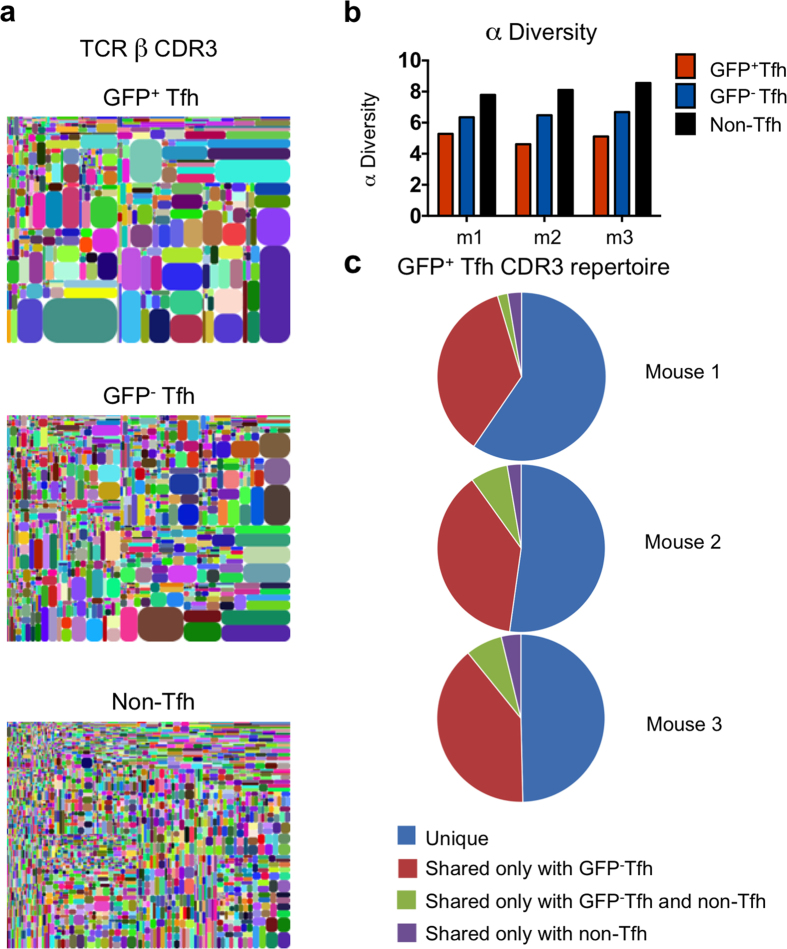
GFP^+^Tfh and GFP^−^Tfh are clonally related. (**a**) Tree maps depicting the clonal complexity of the TCR β repertoire in CD4^+^ T cells from PP. Data are from one of three representative mice. (**b**) α Diversity values of GFP^+^Tfh, GFP^−^Tfh, and non-Tfh CD4^+^ T cells from 3 individual mice (m1–3). (**c**) Overlap of the repertoire of unique TCR Vβ CDR3 sequences from GFP^+^Tfh cells, with the repertoire of GFP^−^Tfh and non-Tfh CD4^+^ cells in the same mouse. Each pie graphic represents all GFP^+^Tfh cells’ unique CDR3 sequences from one mouse (n = 3).

GFP^+^Tfh, GFP^−^Tfh, and GFP^−^CD4^+^ non-Tfh cells were purified from PP of three individual mice from different litters and the VDJ domains of their TCRβ genes were sequenced using RNAseq. All populations displayed broad V β usage (Supplementary Fig. S4). The number of unique CDR3 peptide sequences, as exemplified by tree plots (Fig. 2a), was highest in non-Tfh CD4^+^ cells, intermediate in GFP^−^Tfh cells, and lowest in GFP^+^Tfh cells (Fig. 2a). Analysis of the number of unique TCRβ CDR3 sequences using computational α diversity showed the highest diversity in the repertoire of non-Tfh CD4^+^ cells and the lowest in the repertoire of GFP^+^Tfh cells (Fig. 2b). Comparing repertoires of unique CDR3 peptide sequences revealed that an average of 43% of the repertoire of unique sequences of GFP^+^Tfh cells was shared with the GFP^−^Tfh population of the same mouse, compared to 8% shared with non-Tfh CD4^+^ T cells (Fig. 2c). GFP^−^Tfh cells in turn shared an average of 17% of their repertoire with GFP^+^Tfh cells, and 9% with non-Tfh CD4^+^ T cells of the same mouse (Supplementary Fig. S5a).

We then used β diversity computational analysis of TCRβ CDR3 repertoires to generate a matrix of pair-wise comparisons of the overall repertoire composition of each of the three PP CD4^+^ T cell populations in each mouse (Supplementary Fig. S5b). This analysis takes into account the number as well as the frequency of unique sequences: the maximum pair-wise comparison value of 1 reflects complete repertoire identity, while a value close to zero indicates unrelated repertoires. Comparing GFP^+^Tfh and GFP^−^Tfh populations from the same mouse yielded the highest β diversity values (0.181, 0.158, 0.172). GFP^+^Tfh and GFP^−^Tfh cells exhibited low relatedness with non-Tfh CD4^+^ cells within the same animal (0.012, 0.016, 0.013 and 0.028, 0.044, 0.036, respectively). Pairwise comparisons of cell populations between different animals also yielded low β diversity values (0.004 to 0.035).

The TCRβ repertoire analysis thus showed a high degree of relatedness between GFP^+^Tfh and GFP^−^Tfh cells from PP, suggesting a process of ongoing selection by common antigens and interconversion between the two phenotypes.

### Differentiation of CD4^+^ T cells expressing IL-21 and GFP is driven by TGFβ, IL-6 and RA *in vitro*

We next sought to determine if IL-21/GFP expression could be induced in CD4^+^ spleen cells from IL-21eGFP mice *in vitro* under conditions that mimic the gut microenvironment. IL-6, TGFβ and RA are abundant molecules in the gut that are known to regulate T helper cell differentiation. IL-6 and TGFβ drive Th17 polarization and production of IL-21^37^,^38^, while RA suppresses Th17 differentiation^39^ but not IL-21 production^40^, and allows TGFβ-mediated differentiation of Foxp3^+^ Treg cells^39^.

**Figure 3:**
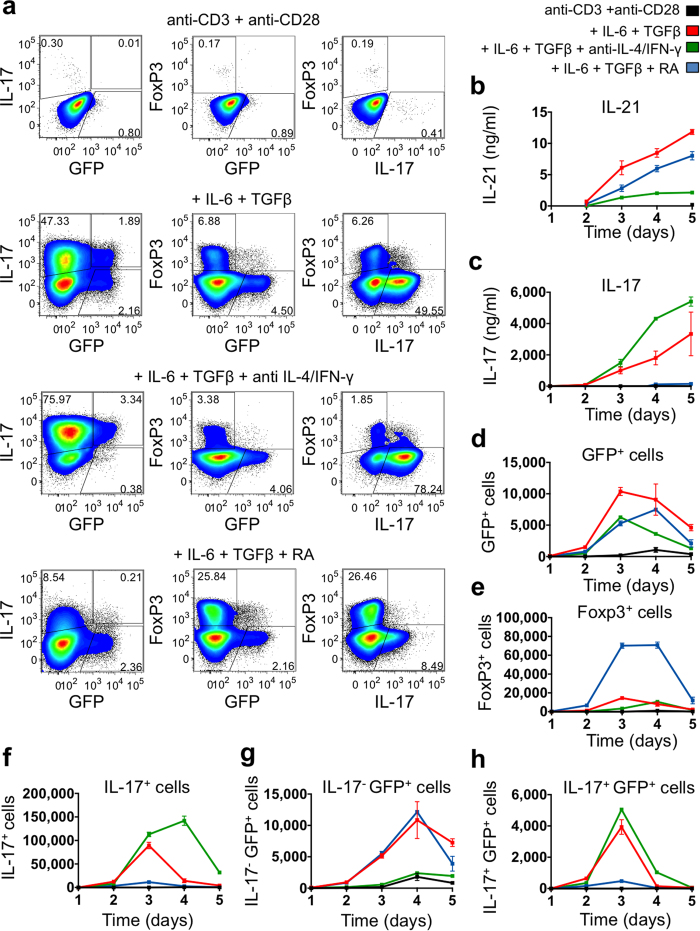
GFP expression is associated with IL-21 production in CD4^+^ cells stimulated *in vitro*. Splenocytes from IL-21eGFP TBmc mice were stimulated for five days with anti-CD3 and anti-CD28 antibodies plus IL-6, TGFβ, neutralizing anti-IL-4 and anti-IFNγ antibodies, and RA, as indicated. Kinetics of cytokine production was determined by intracellular staining and flow cytometry analysis, and by quantification of cytokines in supernatants by ELISA. (**a**) Representative analysis of the expression of GFP, IL-17 and Foxp3 in gated CD4^+^ cells from day 3 post-stimulation. Left panels show plots of IL-17 vs GFP fluorescence, centre panels show Foxp3 vs GFP and right panels show FoxP3 vs IL-17. (**b**) IL-21 levels in culture supernatants. (**c**) IL-17 levels in culture supernatants. (**d**) Numbers of GFP^+^CD4^+^ cells. Numbers of (**e**) Foxp3^+^CD4^+^ cells, (**f**) IL-17^+^CD4^+^ cells, (**g**) IL-17^−^GFP^+^CD4^+^ cells, (**h**) IL-17^+^GFP^+^CD4^+^ cells. (**b–h**) Data are expressed as mean ± s.e.m. of 3 wells per condition, and are representative of two independent experiments.

We thus assessed GFP expression under conditions expected to promote IL-21 production. We used spleen cells from IL-21eGFP TBmc mice as a source of naïve CD4^+^ T cells. All T cells in TBmc mice possess an OVA-specific TCR (DO11.10), and all B cells express a B cell receptor specific for a peptide from hemagglutinin of influenza virus^41^,^42^. T and B cells in TBmc mice remain naïve in the absence of exposure to these specific antigens, and thymic Treg cells are not present. GFP expression was undetectable in untreated IL-21eGFP TBmc mice, and no Tfh, GC or switched B cells were present in spleen (data not shown). Splenocytes from IL-21eGFP TBmc mice were stimulated *in vitro* with anti-CD3 and anti-CD28 alone, or with the addition of IL-6+TGFβ, IL-6+TGFβ+anti-IL-4+anti-IFNγ, or IL-6+TGFβ+RA for 5 days (Fig. 3). The T cell phenotypes were analyzed daily by flow cytometry, and supernatants were collected for quantification of IL-21 and IL-17 (Fig. 3). Splenocytes from TBmc littermates were cultured alongside for comparison (Supplementary Fig. S6).

In anti-CD3 and anti-CD28 stimulated cultures, IL-21 and IL-17 production was negligible (Fig. 3a–c) and few GFP^+^ cells were detected (Fig. 3a,d). In stimulated cultures containing IL-6 and TGFβ (Th17 conditions) approximately 50% of CD4^+^ T cells were IL-17^+^ (Fig. 3a,f and Supplementary Fig. S6a), and 7% of cells were Foxp3^+^IL-17^−^GFP^−^ Tregs (Fig. 3a,e and Supplementary Fig. S6b). Under Th17 conditions, GFP^+^ cells were discernable as two populations, one IL-17^+^ and the other IL-17^−^ (Fig. 3a,g,h). IL-17 and IL-21 were both detected in culture supernatants of cells exposed to IL-6+TGFβ (Fig. 3b,c and Supplementary Fig. S6c,d). Addition of anti-IL-4 and anti-IFNγ antibodies to IL-6+TGFβ cultures further skewed differentiation towards the Th17 phenotype, as described^37^,^38^. Interestingly, the IL-17^−^GFP^+^ population was greatly reduced, and most GFP^+^ cells co-expressed IL-17 (Fig. 3a,g,h).

Addition of RA to IL-6+TGFβ-stimulated cultures led to an expected increase in the frequency of Foxp3^+^ cells (Fig. 3a,e and Supplementary Fig. S6b). It also resulted in a decrease in IL-17^+^ cells and secreted IL-17 protein^39^ (Fig. 3a,c,f and Supplementary Fig. S6a,c). The IL-17^+^GFP^+^ population was also reduced by RA treatment, while the IL-17^−^GFP^+^ population was unaffected (Fig. 3a,g,h). IL-21 levels in supernatants remained high under these conditions, although they did not reach the same levels as in IL-6+TGFβ cultures (Fig. 3b and Supplementary Fig. S6d).

In sum, we demonstrated that GFP^+^ IL-21 producing cells could be induced *in vitro* by culture conditions that favor or suppress co-expression of IL-17.

### Polyclonal T and B cell activation supports differentiation of GFP^+^Tfh cells in Peyers’ Patches

We next asked which type of cellular interactions might be important for differentiation of PP GFP^+^ Tfh cells *in vivo*. Polyclonality of CD4 T and/or B cells could be important to provide a diverse repertoire for antigen recognition. We thus purified polyclonal CD4^+^GFP^−^ T cells from spleens of IL-21eGFP BALB/c mice and transferred the T cells alone or with polyclonal B cells from WT CD45.1 BALB/c mice, into CD45.2 TBmc recipient mice.

As previously described, TBmc mice harbor naive CD4^+^ T cells and B cells that do not react to self or microbial antigens, and provide an environment with normally organized lymphoid tissue.

**Figure 4:**
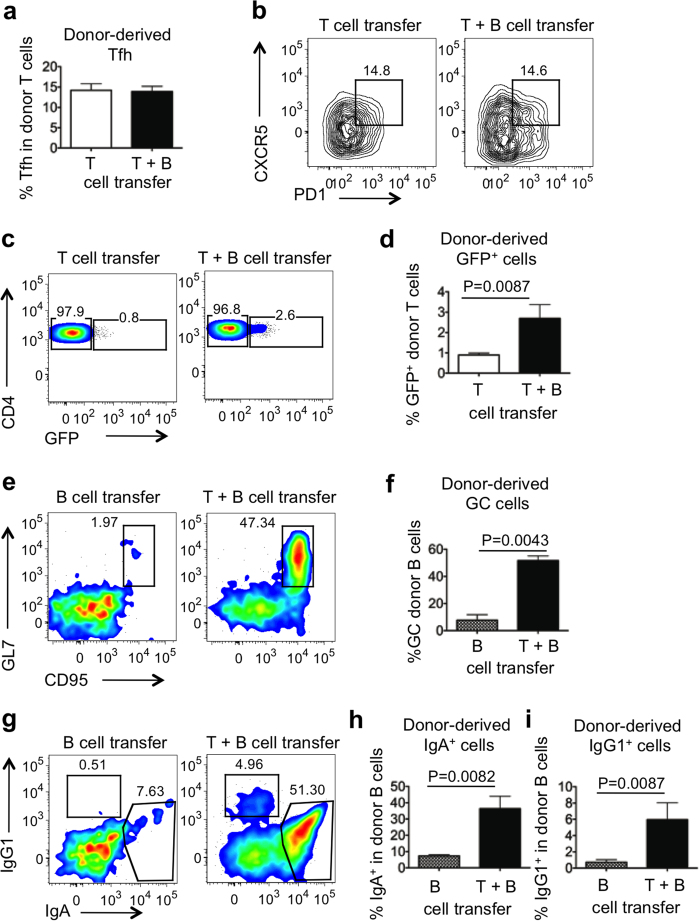
A polyclonal T and B cell repertoire supports the formation of highly differentiated Tfh cells in PP. TBmc (CD45.2^+^) recipient mice were infused with splenic GFP^−^CD4^+^ T cells from IL-21eGFP mice (white bars, T), with GFP^−^CD4^+^ T cells from IL-21eGFP mice and splenic polyclonal B cells from CD45.1^+^ mice (black bars, T+B), or with polyclonal CD45.1^+^ B cells alone (hatched bars, B). 4 weeks post transfer, PP cells were analysed by flow cytometry. (**a**) Percentage of CD4^+^KJ126^−^ donor T cells with CXCR5^+^PD1^+^ Tfh phenotype. (**b**) Representative flow cytometry plots of CXCR5 and PD1 expression in gated CD4^+^KJ126^−^ donor T cells. Numbers in plots indicate the percentage of Tfh cells. (**c**) Representative plots and (**d**) quantification of GFP^+^ cells in gated CD4^+^KJ126^−^ donor T cells in PP. (**e**) Representative plots and (**f**) quantification of the percentage of GC cells within gated B220^+^CD45.2^−^ donor B cells in PP. (**g**) Representative plots and quantification (**h,i**) of the percentage of IgA^+^ and IgG1^+^ cells respectively in gated B220^+^CD45.2^−^ donor B cells in PP. Bar graphs show mean ± s.e.m. of 6–8 mice per group. Data were combined from two independent experiments. Statistical significance was determined by unpaired, two-tailed, Mann-Whitney U-test.

Donor polyclonal CD4^+^ T cells and endogenous T cells were distinguished by staining with the anti-clonotypic antibody KJ1-26, which recognizes endogenous DO11.10 TCR. Donor and recipient B cells were distinguished by expression of CD45.1 and CD45.2, respectively. Four weeks after transfer, differentiation of donor polyclonal T and B cells in PP of recipient TBmc mice was assessed. The frequencies of total donor-derived CD4^+^ T cells (Supplementary Fig. S7a,b) and of donor-derived CD4^+^CXCR5^+^ PD1^+^ Tfh cells (Fig. 4a,b) were comparable in PP of mice that received polyclonal IL-21eGFP CD4^+^ T cells alone or together with polyclonal B cells. However, the percentage of GFP^+^ cells among donor-derived CD4^+^ cells was significantly higher in mice that also received polyclonal B cells (Fig. 4c,d). Donor-derived T cells were detected in MLN and spleen, but they did not express GFP (Supplementary Fig. S7c and data not shown). Recipient T cells (CD4^+^KJ126^+^) did not differentiate into Tfh cells following transfer of either donor polyclonal T cells, or polyclonal T and B cells (Supplementary Fig. S7d).

Donor-derived polyclonal B cell expansion, class switching to IgA and IgG1, and differentiation into GL7^+^CD95^+^ GC cells was evident upon co-transfer of polyclonal CD4^+^ T cells, but was not supported by the recipient OVA-specific CD4^+^ cells alone (Supplementary Fig. S7e,f and Fig. 4e–i). Transfer of polyclonal CD4^+^ T cells also led to the differentiation of a small percentage of endogenous B cells into GC, IgA^+^ and IgG1^+^ B cells (Supplementary Fig. S7e,g,h,i).

We also attempted to induce differentiation of OVA-specific CD4 T cells into GFP^+^Tfh cells in TBmc mice carrying the IL-21eGFP transgene. However, neither immunization with OVA alone (T cell antigen) nor with OVA-HA (T and B cell antigens), by oral or peritoneal routes, induced GFP expression in PP, LN or spleen of TBmc IL-21eGFP mice (data not shown).

In summary, we found that differentiation of polyclonal CD4^+^ T cells into GFP^−^Tfh cells in PP could occur in the presence of HA-specific B cells that do not recognize antigens in the gut, but highly-differentiated GFP^+^Tfh cells could only form in the presence of a polyclonal B cell repertoire. Considering that transplanted T cells spontaneously differentiated into Tfh cells and turned on GFP expression in the PP of recipient mice, we hypothesized that the gut microbiota might provide the adequate source of antigenic stimulation.

### The gut microbiota induces the differentiation of GFP^+^ CD4 T helper cells in Peyers’ Patches of IL-21eGFP mice

**Figure 5:**
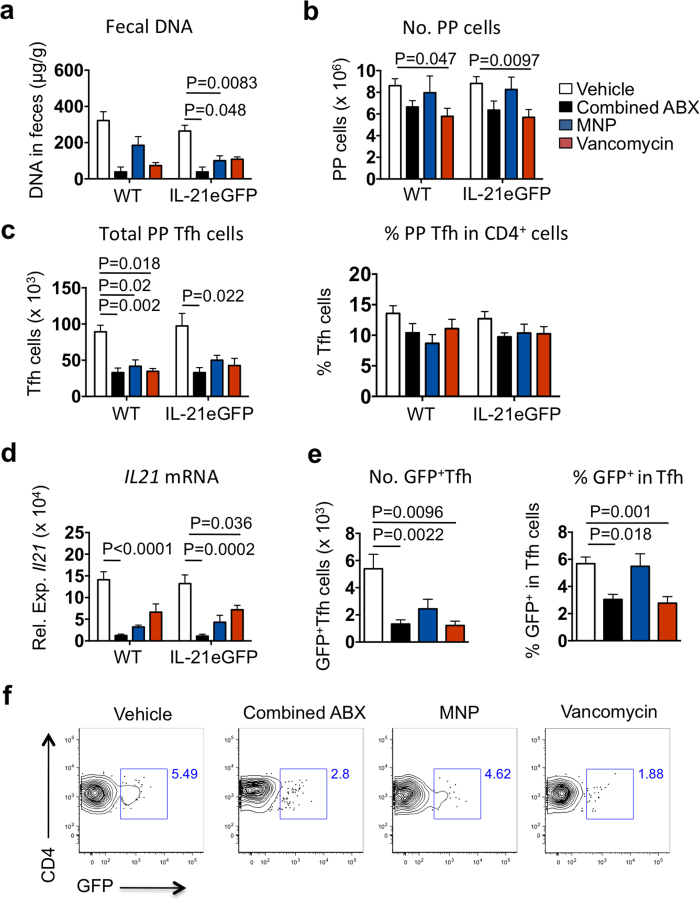
The bacterial microbiota is necessary for the formation of highly differentiated Tfh cells in PP. IL-21eGFP mice and WT littermates were treated with combined broad-spectrum antibiotics (ampicillin, metronidazole, neomycin sulfate and vancomycin; ABX), gram negative-depleting antibiotics (metronidazole, neomycin sulfate and polymyxin; MNP) or vancomycin, from 2 to 6 weeks of age. (**a**) Fecal DNA levels reflect bacterial depletion after antibiotic treatments. (**b**) Total PP cells per mouse at 6 weeks of age. (**c**) Number of Tfh cells per mouse (left graph), and percentage of Tfh cells in total PP CD4^+^ cells (right graph). (**d**) QPCR quantification of *IL21* mRNA levels in PP. (**e**) Number of Tfh cells expressing GFP in PP (left graph) and percentage of GFP^+^ cells in PP Tfh cells. (**f**) Representative flow cytometry plots of GFP expression in gated CD4^+^PD1^+^CXCR5^+^ Tfh cells in the PP of IL-21eGFP mice. Data were combined from three independent experiments (n ≥ 7 mice per group) and are expressed as mean ± s.e.m. Treatment groups within each mouse strain were tested for statistical significance by Kruskal-Wallis followed by Dunn’s multiple comparisons test. P values are shown only for statistically significant differences (p < 0.05).

We then asked whether antibiotic treatments would affect the generation of GFP^+^Tfh cells in IL-21eGFP mice. Mice were treated with either a broad spectrum antibiotic combination (ABX treatment- ampicillin, metronidazole, neomycin sulfate and vancomycin), a gram-negative bacteria depleting antibiotic mix (MNP treatment- metronidazole, neomycin sulfate and polymyxin B) or with vancomycin alone to remove gram-positive bacteria^29^,^30^. All three treatments resulted in a lower amount of bacterial DNA in feces of both IL-21eGFP and wild type (WT) mice, though the differences were statistically significant only for the ABX and MNP treatments of IL-21eGFP mice (Fig. 5a). Changes in total PP cell numbers were only significantly lower in vancomycin treated mice (Fig. 5b). There was a reduction of PP Tfh cells in mice of all treatment groups, but only in the combined ABX treatment was the reduction statistically significant in both WT and IL-21eGFP mice (Fig. 5c, left graphic). The frequency of Tfh cells among CD4^+^ T cells was not significantly changed by any antibiotic treatment (Fig. 5c, right graphic). QPCR analysis demonstrated a significant reduction in IL-21 mRNA levels following the ABX treatment in both WT and IL-21eGFP mice (Fig. 5d). Interestingly, the absolute number and the % of GFP^+^Tfh among Tfh cells were significantly reduced by the combined ABX treatment and by vancomycin in IL-21eGFP mice (Fig. 5e,f). These results demonstrate that the microbiota is necessary for the induction of intestinal GFP^+^ Tfh cells in IL-21eGFP mice.

### Mucosal expression of GFP and IL-21 in IL-21eGFP mice begins around 4 weeks of age

Commensal bacteria colonize the gut during lactation and quickly expand after weaning, leading to great expansion of mucosal lymphoid follicles and formation of germinal centers. Given the microbiota requirement for the existence of highly-differentiated Tfh cells in PP, we performed a longitudinal study (from weaning age to 8 weeks) to determine the age at which PP IL-21/GFP^+^ Tfh cells appeared. In addition, we addressed the correlation between GFP expression and B cell responses in PP.

**Figure 6:**
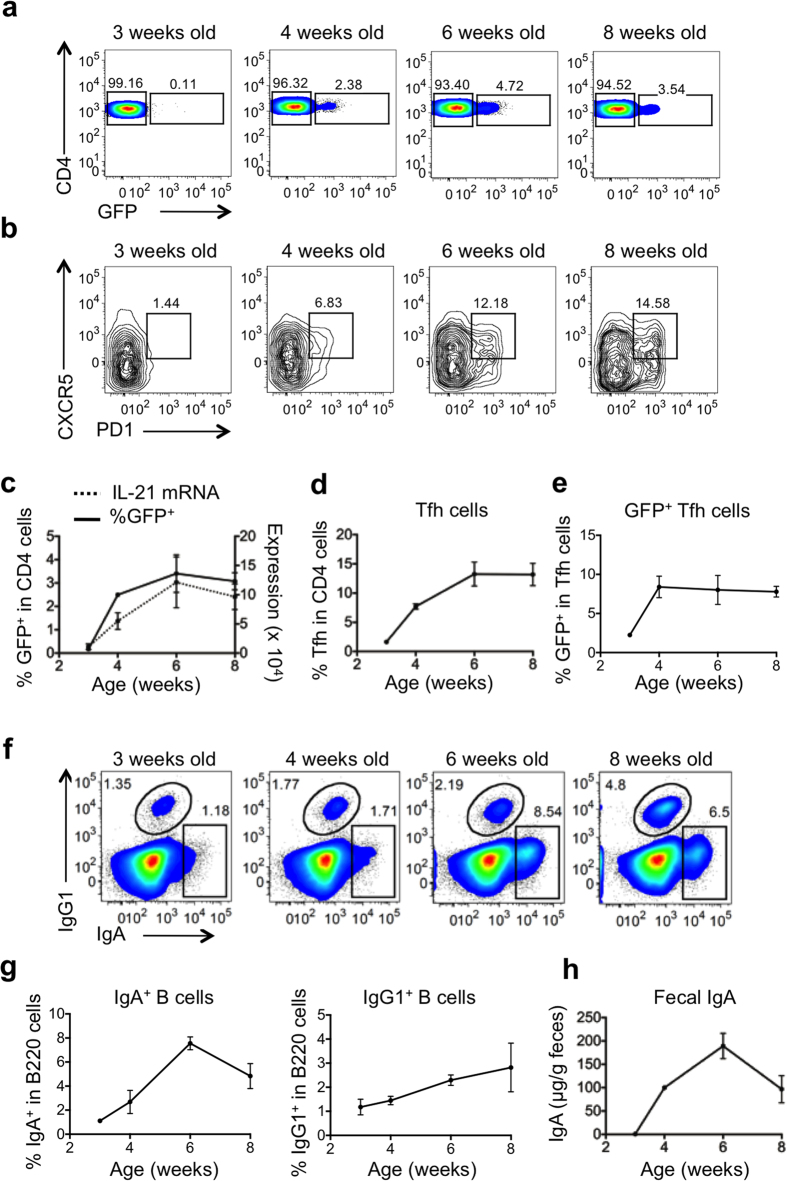
PP Tfh cells, GC and mucosal antibody responses develop with age in IL-21eGFP reporter mice. PP cells and fecal pellets were collected from 3, 4, 6 and 8 week old IL-21eGFP mice and analysed. (**a**) Representative flow cytometry plots of GFP expression in gated CD4^+^ cells. (**b**) Representative flow cytometry plots of gated CD4^+^ cells show appearance of CXCR5^+^PD1^+^ Tfh cells in PP with age. (**c**) Percentage of CD4^+^ cells expressing GFP (solid line), and QPCR quantification of *IL21* mRNA levels in PP (dotted line). (**d**) Percentage of CXCR5^+^PD1^+^ Tfh cells in PP CD4^+^ cells. (**e**) Percentage of PP Tfh cells expressing GFP. (**f**) Representative flow cytometry plots showing IgA^+^ and IgG1^+^ cells in gated PP CD19^+^ cells. (**g**) Quantification of the percentage of IgA^+^ and IgG1^+^ cells in PP CD19^+^ cells. (**h**) IgA levels in fecal extract supernatants as measured by ELISA. Data are expressed as mean ± s.e.m of 3–8 mice per group and are representative of two independent experiments.

We observed GFP expression in CD4^+^ T cells from PP shortly after weaning, at about 4 weeks of age, increasing to maximum levels at 6 weeks of age. GFP expression appeared concomitant with Tfh cell appearance and IL-21 mRNA production in PP (Fig. 6a–e). Likewise, IgA^+^ and IgG1^+^ B cells in PP, and fecal IgA, were all first detectable around 3 to 4 weeks of age (Fig. 6f–h). Therefore, the appearance of GFP^+^CD4^+^ cells correlated with the development of Tfh cells, IgA and IgG1 B cells in PP.

### Ablation of GFP^+^ cells in IL-21eGFP mice leads to reduction of GC and IgG1^+^ cells in Peyers’ Patches and to shifts in microbiota composition

IL-21eGFP mice express the diphtheria toxin (DT) receptor under control of the IL-21 promoter (Supplementary Fig. S1a), allowing DT-mediated deletion of GFP-expressing cells. To assess the functional impact of the absence of GFP^+^ Tfh cells, IL-21eGFP mice were treated with DT for 3 weeks, starting at 3 weeks of age. DT treatment prevented the emergence of GFP^+^CD4^+^ cells in young mice (Supplementary Fig. S8a), which otherwise occurred from 4 weeks of age (Fig. 6). DT treatment did not significantly affect the percentage or number of Tfh cells in PP (Supplementary Fig. S8b). The PP cellularity, the number of CD4^+^ T cells and of B220^+^ B cells, were also comparable between WT and IL-21eGFP mice that were treated with DT or PBS control (Supplementary Fig. S8c–e).

**Figure 7:**
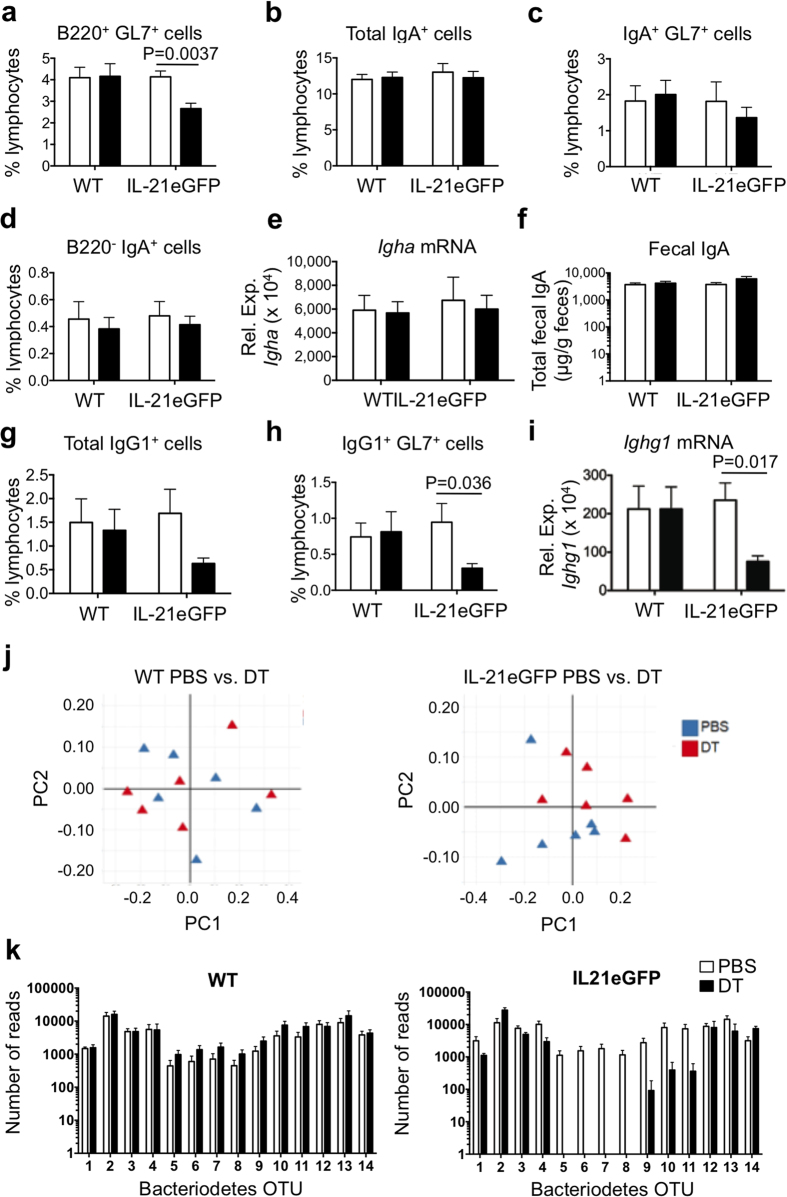
DT depletion of GFP^+^ cells in IL-21eGFP mice impacts GC in PP and gut bacteria diversity. IL-21eGFP mice and WT littermates were administered DT from 3–6 weeks of age. PP cells, total PP RNA, feces and serum were harvested at 6 weeks of age and analyzed. (**a**) Percentage of B220^+^GL7^+^ GC cells in PP lymphocytes. (**b**) Percentage of total IgA^+^ cells, (**c**) IgA^+^GL7^+^ GC cells and (d) B220^−^IgA^+^ cells in PP lymphocytes. (**e**) QPCR analysis of *Igha* mRNA expression in PP. (**f**) Levels of fecal IgA. (**g**) Percentage of total IgG1^+^ cells and (**h**) IgG1^+^GL7^+^ GC cells in PP lymphocytes. (**i**) QPCR analysis of *Ighg1* mRNA expression levels in PP. (**a–i**) Bar graphs show mean±s.e.m. of 7–12 mice per group from two independent experiments. Statistical significance was determined by unpaired, two-tailed, Mann-Whitney U-test. P values are shown only for statistically significant differences (p < 0.05). (**j**) Principal component analysis (PCA) of β diversity of the gut microbiome from WT (left panel) and IL-21eGFP (right panel) fecal samples of individual mice treated with PBS (blue triangles) or DT (red triangles). (**k**) Bacteriodetes OTUs with significantly different abundance in PBS vs DT treated IL-21eGFP mice. Number of OTU reads from fecal microbiome 16S DNA sequencing of WT (left panel) and IL-21eGFP (right panel) mice that were treated with PBS (clear bars) or DT (black bars). Bacteriodetes OTUs analysed: 1 - AY975430.1.1357, 2 - EU655718.1.1320, 3 - EF603688.1.1483, 4 - AATE01000019.1017.2438, 5 - EF099203.1.1405, 6 - EF098805.1.1411, 7 - EF406418.1.1506, 8 - EU455595.1.1410, 9 - EU455438.1.1412, 10 - EF406534.1.1514, 11 - EF098751.1.1405, 12 - EU790981.1.1497, 13 - EU511199.1.1411, 14 - EU509932.1.1403.

Interestingly, ablation of GFP^+^Tfh resulted in a significant reduction in the percentage of GC B cells in IL-21eGFP mice but not in WT mice (Fig. 7a). No changes were detected in the frequency of total IgA^+^ B cells, GC IgA^+^ B cells or B220^−^IgA^+^ B cells following DT treatment of IL-21eGFP mice (Fig. 7b–d). Likewise, neither the levels of IgA mRNA in PP (Fig. 7e) nor fecal IgA levels appeared affected (Fig. 7f). DT treatment of IL-21eGFP mice resulted in average lower numbers IgG1^+^ B cells in PP, though the change was not statistically significant (Fig. 7g). However, IgG1^+^ GC B cells and total IgG1 mRNA levels in PP were significantly decreased (Fig. 7h,i). Total IgG1 and IgA serum levels did not change following DT treatment of WT or IL-21eGFP mice (Supplementary Fig. S8f,g), nor did the expression of other antibody isotypes in PP (Supplementary Fig. S8h–j).

To determine if the depletion of the GFP^+^ T cells may have affected the composition of the gastrointestinal bacteria, we carried out 16S ribosomal RNA sequencing of DNA isolated from stool samples of DT or PBS treated IL-21eGFP mice. Principal component analysis (PCA) of the fecal bacteria composition demonstrated a dissociation of DT vs PBS treated samples of IL-21eGFP mice but not in WT control mice (Fig. 7j). When the bacteria composition was analyzed using β diversity computational analysis, a significant reduction in diversity due to DT treatment was found in IL-21eGFP mice (p-value 0.027) but not WT mice (p-value 0.521). A similar trend was observed for α diversity analysis but the difference was not statistically significant (Supplementary Fig. S9a). There were no significant changes in bacterial composition at the family level (Supplementary Fig. S9b), however fecal samples from DT treated IL-21eGFP mice, but not WT control mice, had a significant reduction in a number of *Bacteriodetes* operational taxonomic units (OTU’s) in comparison with PBS treated controls (Fig. 7k).

Thus, the absence of GFP^+^ Tfh cells in young IL-21eGFP mice led to a reduction in GC B cells and IgG1^+^ B cells in PP and to shifts in the composition of the gastrointestinal bacteria, indicating that the highly differentiated T helper cells of PP play a role in gut homeostasis.

## Discussion

Tfh cells are essential for the GC reaction and for development of high affinity antibodies^1^,^2^,^43^. Their secretion of IL-21 promotes GC B cell proliferation and survival, immunoglobulin class switching and plasma cell differentiation^15^,^44^,^45^. To understand the *in vivo* expression patterns of IL-21 and the function of IL-21-expressing cells we developed a new BAC transgenic IL-21 reporter mouse strain expressing *DTR-eGFP* under the control of *IL21* regulatory sequences (IL-21eGFP). GFP expression in IL-21eGFP transgenic mouse strain marks a subpopulation of Tfh cells in PP. GFP was not expressed by spleen Tfh cells *in vivo*, indicating that the GFP^+^Tfh population is specific to the gut. Our studies showed that this subpopulation is induced by the microbiota and is necessary for optimal GC reactions in PP.

Compared to PP GFP^−^Tfh cells, PP GFP^+^Tfh cells expressed higher levels of several the Tfh-associated molecules, such as Bcl6, CXCR5, IL-21, and lower levels of molecules that are downregulated during Tfh differentiation, including CCR7 and CD127/IL7rα. Bcl6, CXCR5, and IL-21 are all integral to the differentiation and function of Tfh cells^11^,^43^,^46^,^47^. The higher expression of Bcl6 by GFP^+^Tfh cells than by GFP^−^Tfh cells suggest a more polarized phenotype in the former subset, as Bcl6 is the master transcription factor of Tfh cells^7^,^8^,^48^. Higher expression of CXCR5 and lower expression of CCR7 in GFP^+^ Tfh cells compared with GFP^−^Tfh cells predicts stronger chemotactic response to CXCL13 of GFP^+^Tfh cells, and preferential localization to follicles and the LZ of the GC^3^,^6^,^10^. Moreover, GFP^+^ Tfh cells exhibited the most polarized expression of the activating receptors CD226 and CD96, which are dowregulated in Tfh cells, and of the inhibitory receptor TIGIT, which is upregulated in Tfh cells. CD226, CD96 and TIGIT are all ligands of the poliovirus receptor (PVR/CD155). TIGIT^+^ human Tfh cells have been shown to have enhanced B-cell helper function compared to TIGIT^−^ Tfh cells^49^, and the TIGIT receptor CD155 was found expressed in a subset of human follicular dendritic cells (FDC), suggesting interactions of TIGIT^+^ Tfh cells with CD155^+^ FDC^50^. Thus, the population of Tfh cells identified by GFP expression in PP of IL-21eGFP mice is highly differentiated and exhibits a phenotype suggestive of a capacity for cellular interactions in the GC.

The differences between GFP^+^Tfh and GFP^−^Tfh cell populations from PP led us to ask whether the two populations arose independently or had a common clonal origin. One indication of clonal relatedness is the degree of TCR repertoire overlap: we found that more than 40% of the unique TCRβ CDR3 sequences from GFP^+^Tfh cells were contained within the larger GFP^−^Tfh repertoire. Statistical analysis confirmed that the GFP^+^Tfh and GFP^−^Tfh^+^ cell TCRβ repertoires in each mouse were highly related. It is therefore most likely that GFP^+^Tfh cells arise from a GFP^−^Tfh^+^ precursor and represent a more advanced stage of Tfh cell differentiation.

*In vitro*, IL-21/GFP expression was induced in spleen cells from IL-21eGFP mice by TCR- and CD28- stimulation in the presence of IL-6 and TGFβ, conditions that promote Th17 differentiation^9^,^37^. The addition of RA suppressed IL-17 production but not IL-21 production, in agreement with previous studies^39^,^40^ and yielded an IL-21/GFP^+^ cell population that did not express IL-17 or Foxp3. The *in vivo* analysis of IL-21/GFP expressing CD4 cells demonstrated that these cells are induced by factors present in the gut environment, including IL-6, TGFβ and RA. Thus, the *in vitro* conditions for IL-21/GFP expression recapitulate to some degree the intestinal inductive environment, while the microenvironment in the spleen of immunized mice did not provide the necessary signals for differentiation of GFP expressing CD4 cells.

Although these studies did not explore the conversion of Th17 or Treg cells into IL-21/GFP^+^ T helper cells, they are not incompatible with previous reports demonstrating the plasticity of Tregs^51^ and Th17^52^ cells in the mucosal environment, specifically their ability to convert to Tfh cells within PP.

Several reports demonstrate the importance of cognate T-B interactions in maintaining the Tfh response^53^, however there is evidence that Tfh cells could form in mice where these interactions were impaired^54^. Moreover, there is doubt concerning the universal need for antigen-specific B cell recognition and T-B interactions in the development of GC^20^,^55^,^56^. We carried out adoptive transfer experiments to understand the nature of the T-B interactions driving the development of GFP^+^Tfh cells in PP. Infusing polyclonal splenic GFP^−^CD4^+^ T cells from IL-21eGFP mice into TBmc mice, which have naïve monospecific T and B cell repertoires, resulted in the differentiation of CXCR5^+^PD-1^+^CD4^+^ cells from donor cells. However, the highly differentiated GFP^+^Tfh cell population did not form unless polyclonal B cells were co-transferred, suggesting that Tfh differentiation requires antigen-specific cognate interactions of T and B cells. A possible explanation is that polyclonal B cells provided a large repertoire containing clones that recognize intestinal antigens, and would effectively present the intestinal antigens to CD4 clones through cognate interactions. In contrast, monospecific B cells from TBmc mice do not bind intestinal antigens, and would not engage efficiently with intestinal-antigen specific CD4 cells.

To determine whether the gut microbiota was the source of antigenic stimulation we used various antibiotic treatments to deplete gastrointestinal bacteria populations and assess the impact on GFP^+^Tfh cells. Broad-spectrum antibiotic treatment significantly reduced levels of IL-21 in PP. However, depletion of Vancomycin-sensitive bacteria had the greatest negative impact on the differentiation of GFP^+^Tfh cells, suggesting a role for gram positive bacteria. Innate recognition of bacteria by DCs and FDCs may be the mechanisms whereby IL-21 production by Tfh cells is induced in PP, analogous to the process in Th17 cells^57^. Direct sensing of microbial products by CD4^+^ T cells may also be necessary, as demonstrated previously for microbiota-induced Tregs^58^. Further investigation will be required to define the precise link between bacteria and the induction of highly differentiated Tfh cells in PP, and to account for the role of polyclonal B cells.

GFP^+^CD4^+^ T cells and IL-21 production in PP of IL-21eGFP mice were first detected at 3–4 weeks of age, coincident with increased mucosal IgA and IgG1, and with weaning. Thus, mucosal immune responses in the IL-21eGFP reporter mice are supported by an established gut microbiota, which is well known to develop with age in both mice and humans.

The inclusion of a DTR tag in IL-21eGFP mice enabled the specific depletion of GFP expressing cells to assess their function. Preventing the establishment of GFP^+^Tfh cells in the PP of young mice led to a significant reduction in PP GC B cells and GC IgG1 cells, confirming the importance of IL-21-producing Tfh cells in driving the GC reaction and the production of IgG1^15^,^44^. There were no changes in serum IgG1 concentration in DT treated IL-21eGFP mice. This suggests that IgG1 cells that differentiate in PP do not give rise to the majority of serum IgG1. No changes were detected in IgA cell number in PP, or in the levels of fecal IgA levels when GFP^+^ T helper cells were ablated. There were also no changes detected in the production of IgG2a, IgG2b or IgG3 in PP. Production of IgA in the gut occurs through T-dependent and independent mechanisms^20^,^59^,^60^ and IL-21 has been implicated in the production of mucosal IgA^13^. It is thus possible that GFP^−^Tfh cells, which are less differentiated than GFP^+^Tfh cells, provided enough IL-21 to maintain normal production of IgA, or that IgA levels were maintained through compensatory T cell independent mechanisms. This may also apply to other immunoglobulin isotypes.

Another possibility is that ablation of the highly differentiated PP Tfh cells did result in some changes in IgA production that were not detected in our assays, but might have been responsible for the described changes in bacteria composition. Future in depth studies of immunoglobulin repertoire in the presence or absence of the GFP^+^ CD4 T cells will determine if these cells regulate antibody specificity and affinity.

The decrease in IgG1^+^ PP cells after GFP^+^ cell ablation is compatible with the fact that most IgG1^+^ cells in PP have a GC phenotype, and GFP^+^ T cell ablation decreased GC cells in this location. In addition, IgG1 is particularly dependent on IL-21, as it is the most decreased immunoglobulin in IL-21 or IL-21R deficient mice^15^,^44^. Unlike IgA, IgG1 does not readily traverse the epithelium, except during inflammation^61^,^62^, therefore its role in the gut is poorly understood^19^,^20^,^62^. However, 3–4% of human lamina propria B cells are IgG^+^ and the majority recognizes antigens from gut bacteria^19^,^63^. In addition, as some bacteria can bind to the surface of, and perhaps enter PP^64^, IgG1 could interact with these microbes directly within the mucosal tissue. The production of the secretory J chain by IgG positive cells would allow transport of IgG antibody into the lumen^62^,^65^. The function of IgG against intestinal pathogenic bacteria has only recently started to be unveiled^17^,^18^. Antigen-specific IgG was shown to be required for neutrophil elimination of virulent intestinal *Citrobacter rodentium*^17^, and IgG against enteric *E. coli* and *Salmonella* protected mice from systemic infections by these pathogens^18^.

In addition to the effects on PP B cells, we report that GFP^+^ T cell depletion in IL-21eGFP mice resulted in a small but significant reduction in the overall diversity of the gut microbiome and a specific decrease in some *Bacteriodetes* OTUs. This suggests that, in addition to the aforementioned requirement of commensal bacteria for induction of GFP^+^ T helper cells, these cells reciprocally regulate the intestinal bacteria.

In summary, using a new IL-21eGFP reporter mouse strain we identified a subpopulation of highly differentiated T helper cells in PP of the gut that are induced by intestinal bacteria and regulate GC and IgG1 responses in PP. We have thus generated an experimental system that can facilitate the identification of the specific antigenic determinants, cellular interactions and environmental factors involved in the induction of highly differentiated intestinal Tfh cells. This can lead to a better understanding of the homeostatic relationships between the adaptive immune system and the indigenous intestinal bacteria.

## Methods

### Mice

IL-21eGFP transgenic mice were generated by BAC technology (Supplementary Methods) and crossed to BALB/c and TBmc mice (also in BALB/c background)^41^. Heterozygous IL-21eGFP transgenic mice were bred with non-transgenic mice to generate litters containing transgenic and non-transgenic pups. Mice were bred and housed under specific pathogen-free conditions at the Skirball Institute Animal Facility (New York University School of Medicine; NYUSM) or the Biological Resource Centre (BRC; A*STAR, Singapore). Mice were fed Harlan 2918 diet and water *ad libitum*,. At weaning time, mice were separated by gender and housed in groups of 2–5 mice/cage. The mice were genotyped by PCR on tail DNA as described in Supplementary Methods. All animal procedures were approved by the Institutional Animal Care and Use Committees of the NYUSM and BRC/A*STAR, and were carried out in accordance with the approved guidelines. Unless otherwise stated, gender matched 6–8 week old male or female mice were used in all experiments.

### Adoptive transfer experiments

Spleens were pooled from 4–6 donor mice for purification of CD4^+^ T cells or B cells. To isolate CD4^+^ cells from the pooled spleens of IL-21eGFP mice, B cells were removed using anti-B220 microbeads (Miltenyi Biotech). The remaining cells were incubated with APC-anti-CD4, eFluor450-anti-CD19, PE-anti-CD8, PE-anti-CD11c, PE-anti-CD25, PE-anti-CD49b, PE-anti-CD326, PE-anti-Ly76 and PE-anti-MHCII antibodies. The GFP^−^ T cells were isolated as the PE^−^CD4^+^GFP^−^ population by fluorescence-activated cell sorting in a FACSARIA apparatus (BD Biosciences). B cells for transfer were isolated from pooled spleens of CD45.1 BALB/c mice using microbeads (Miltenyi Biotech). The cell suspension was first enriched in B220^+^ cells by depletion of CD4^+^ T cells, and then positively sorted using anti-B220 microbeads. 5 × 10^5^ purified polyclonal CD4^+^ T cells, 2 × 10^7^ polyclonal B cells, or a combination of both, were injected i.v. into the tail veins of TBmc recipient mice. The differentiation of Tfh cells and IgG1 and IgA-producing cells in lymphoid organs was analyzed four weeks later.

### *In vitro* splenocyte culture

Splenocytes from IL-21eGFP and WT mice were seeded at 8 × 10^5^ cells in 0.2 ml/well into 48 well plates and stimulated with anti-CD3 (5 μg/ml) and anti-CD28 (1 μg/ml) antibodies (BD Biosciences). The cytokines IL-6 (20 ng/ml; R & D Systems) and TGFβ (5 ng/ml; BioLegend), anti-IL-4 antibodies (10 μg/ml; BioLegend), anti-IFNγ antibodies (10 μg/ml; BioLegend), and all trans retinoic acid (RA) (10 nM; Sigma) were added at the beginning of the cultures in various combinations (IL-6 alone; IL-6+TGFβ; IL-6+TGFβ+anti-IL-4+anti-IFNγ; IL-6+TGFβ+RA). Cells were cultured for 5 days and culture samples taken for analysis daily. The expression of GFP, Foxp3, and IL-17 were determined by intracellular staining, and the levels of IL-21 and IL-17 in culture supernatants were determined by ELISA.

### Statistics

Statistical significance was determined by unpaired, two-tailed, Mann Whitney U-test for comparison of two groups with one variable. Where more than two variables were being compared, Kruskal-Wallis was applied followed by Dunn’s post-hoc analysis to obtain individual p values. Differences with p value < 0.05 were considered statistically significant. Results are presented as mean ± s.e.m. All analyses were carried out using Prism 5 (GraphPad Software). Statistical analysis of microarray hybridization, TCRβ sequencing and 16S bacteria RNA sequencing are described in Supplementary Methods.

## Additional Information

**How to cite this article**: Jones, L. *et al.* A subpopulation of high IL-21-producing CD4^+^ T cells in Peyer’s Patches is induced by the microbiota and regulates germinal centers. *Sci. Rep.* **6**, 30784; doi: 10.1038/srep30784 (2016).

## Supplementary Material

Supplementary Information

Supplementary Dataset 1

Supplementary Dataset 2

Supplementary Dataset 3

Supplementary Dataset 4
