# Corrigendum: A subpopulation of high IL-21-producing CD4^+^ T cells in Peyer’s Patches is induced by the microbiota and regulates germinal centers

**DOI:** 10.1038/srep34899

**Published:** 2016-10-10

**Authors:** Leigh Jones, Wen Qi Ho, Sze Ying, Lakshmi Ramakrishna, Kandhadayar G. Srinivasan, Marina Yurieva, Wan Pei Ng, Sharrada Subramaniam, Nur H. Hamadee, Sabrina Joseph, Jayashree Dolpady, Koji Atarashi, Kenya Honda, Francesca Zolezzi, Michael Poidinger, Juan J. Lafaille, Maria A. Curotto de Lafaille

Scientific Reports 6: Article number: 30784; 10.1038/srep30784 published online: 08 08 2016; updated: 10 10 2016.

In the Supplementary Information file originally published with this Article there was an error in Supplementary Figure 1b, where the y-axis “GFP” and x-axis “CD4” were inverted. These errors have been corrected in the Supplementary Information that now accompanies the Article.

