# Supplementary material for: A subpopulation of high IL-21-producing CD4+ T cells in Peyer’s Patches is induced by the microbiota and regulates germinal centers
Source: Sci Rep. 2016 Aug 8;6:30784. doi: 10.1038/srep30784 (PMC4976330; doi:10.1038/srep30784)
Supplement: Supplementary Information [file srep30784-s1.pdf]

SUPPLEMENTARY MATERIALS

A subpopulation of high IL-21-producing $CD4^+$ T cells in Peyer's Patches is induced by the microbiota and regulates germinal centers

Leigh Jones, Wen Qi Ho, Sze Ying, Lakshmi Ramakrishna, Kandhadayar G. Srinivasan, Marina Yurieva, Wan Pei Ng, Sharrada Subramaniam, Nur H. Hamadee, Sabrina Joseph, Jayashree Dolpady, Koji Atarashi, Kenya Honda, Francesca Zolezzi, Michael Poidinger, Juan J. Lafaille, Maria A. Curotto de Lafaille

SUPPLEMENTARY FIGURES

SUPPLEMENTARY METHODS

SUPPLEMENTARY FIGURES

Supplementary Figure S1

Generation and characterization of IL-21eGFP mice.

Supplementary Figure S2

$GFP^+CXCR5^+PD1^+$ $CD4^+$ T cells express a polarized Tfh phenotype.

Supplementary Figure S3

$GFP^+CXCR5^+PD\text{-}1^+CD4^+$ cells are highly differentiated Tfh cells.

Supplementary Figure S4

GFP^+Tfh and GFP^-Tfh cells from PP have a diverse polyclonal TCRβ repertoire.

Supplementary Figure S5

The TCRβ CDR3 repertoire of PP $CD4^+$ T cell subsets

Supplementary Figure S6

Induction of IL-21 expression in $CD4^+$ T cells activated in the presence of TGFβ, IL-6 and RA.

Supplementary Figure S7

Splenic GFP^-CD4^+ T cells IL-21eGFP mice traffic to PP where they differentiate into GFP^+Tfh cells and drive B cell activation.

Supplementary Figure S8

DT depletion of GFP^+ cells in IL-21eGFP mice results in alterations of local B cell activation and antibody production in the PP.

Supplementary Figure S9

Overall composition of gut microbiome was similar between WT and IL21eGFP mice.

Supplementary Figure S1

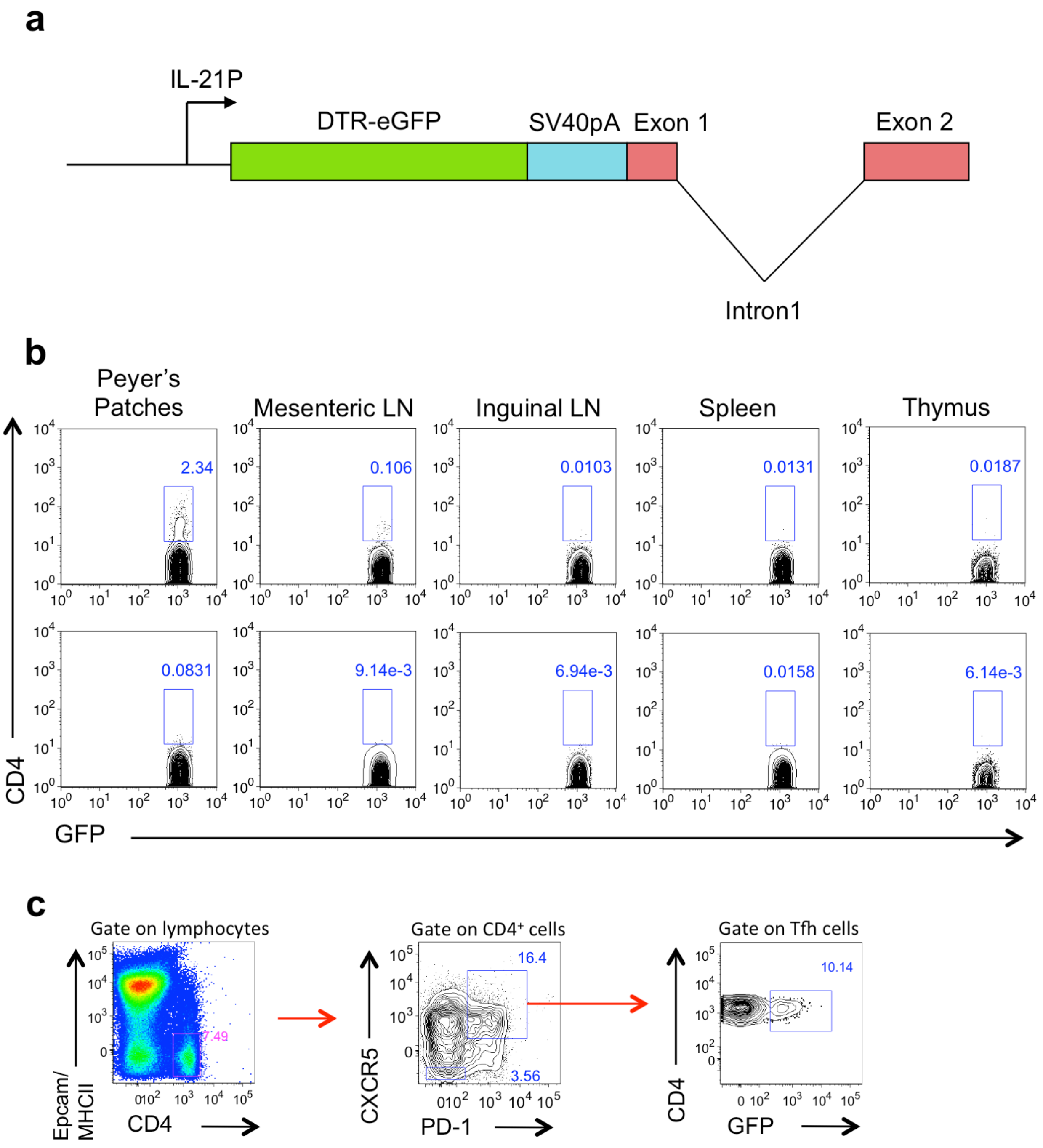

Supplementary Figure S1 Generation and characterization of IL-21eGFP mice.

(a) Schematic representation of the IL-21eGFP BAC transgenic construct. The *DTR-eGFP-SV40pA* gene cassette was inserted downstream of the IL-21 promoter (IL-21P), leaving intact splicing signals and intron 1 sequences. (b) Representative flow cytometry

analysis of GFP expression within the $CD4^+$ cell population from Peyer's Patches, mesenteric lymph node, inguinal lymph node, spleen and thymus of IL-21eGFP transgenic mice (upper row) and non-transgenic littermates (lower row). (c) Gating strategy for analyzing GFP expression in $CD4^+$ Tfh cells. Complements Figure 1 of the manuscript.

Supplementary Figure S2

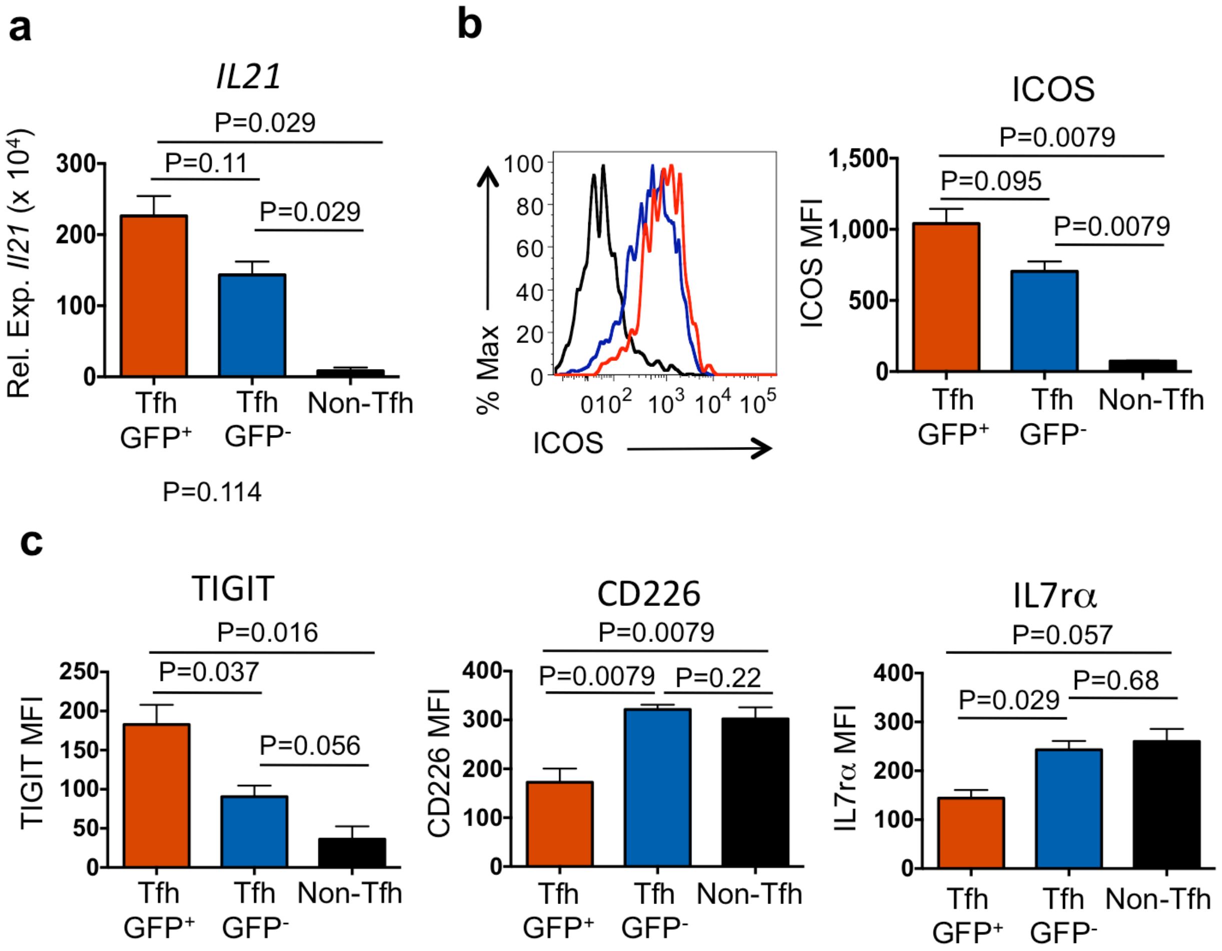

Supplementary Figure S2 $GFP^{+}CXCR5^{+}PD1^{+}$ $CD4^{+}$ T cells express a polarized Tfh phenotype. (a) QPCR analysis of *IL21* mRNA expression in sorted PP $CD4^{+}$ cell populations from IL-21eGFP mice (data from three independent sorting experiments). (b) Representative flow cytometry histograms (left) and quantification of ICOS levels by MFI (right) in gated $GFP^{+}CXCR5^{+}PD1^{+}$ (GFP^{+}Tfh), $GFP^{-}CXCR5^{+}PD1^{+}$ (GFP^{-}Tfh), and $GFP^{-}CXCR5^{-}PD1^{-}$ (Non-Tfh) $CD4^{+}$ populations from PP (n=5). (c) MFI levels of TIGIT (left), CD226 (center) and IL-7rα (right) determined by flow cytometry in gated GFP^{+}Tfh, GFP^{-}Tfh, and GFP^{-} Non-Tfh $CD4^{+}$ populations from PP (n=3). The data in (c) is representative of two experiments. Statistical significance was determined by unpaired, two-tailed, Mann-Whitney U-test. Complements Figure 1 of the manuscript.

Supplementary Figure S3

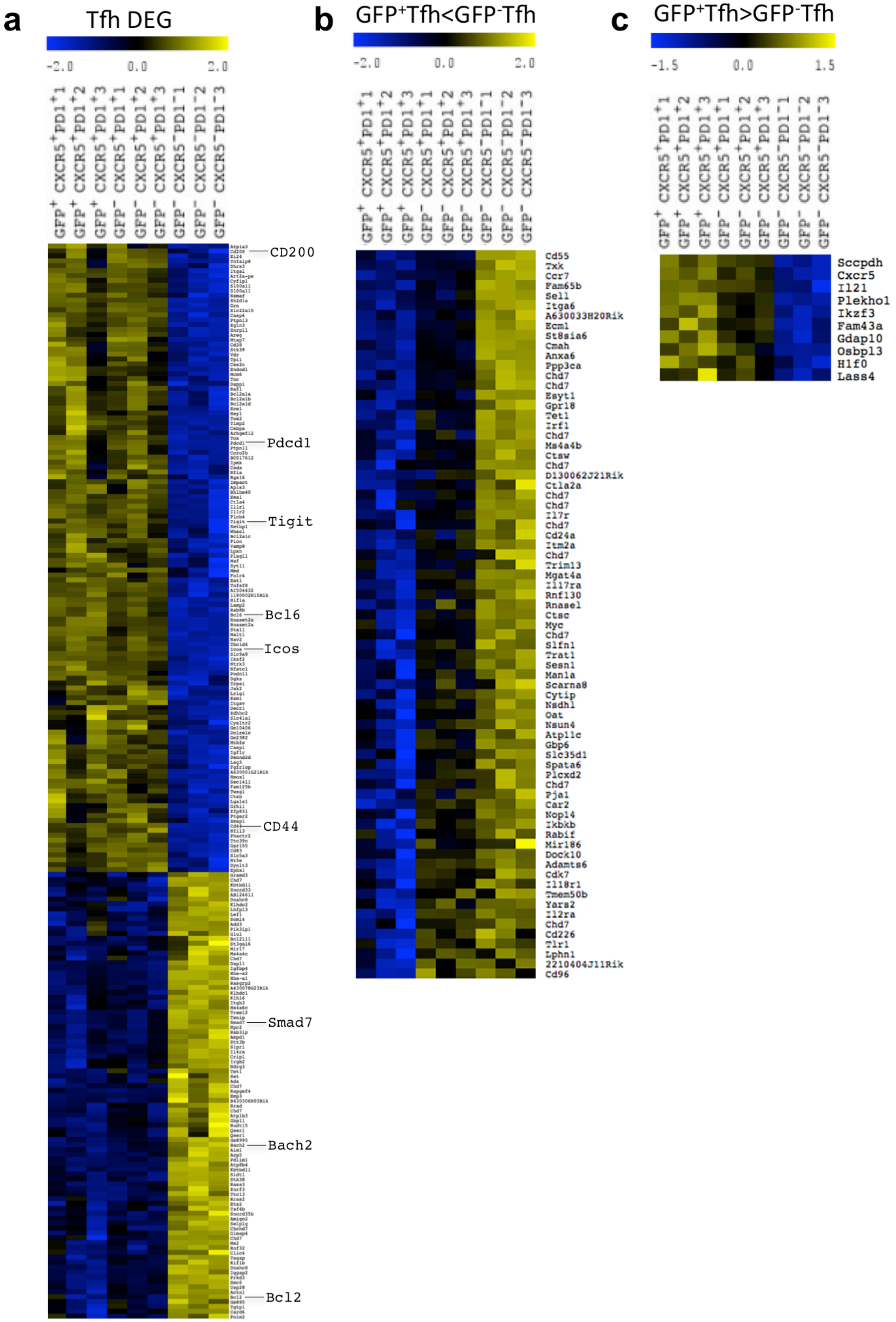

Supplementary Figure S3 $GFP^+CXCR5^+PD\text{-}1^+CD4^+$ cells are highly differentiated Tfh cells. (a-c) Heatmap representation of differentially expressed genes (DEG) in Tfh cells, identified by comparison between all $CXCR5^+PD\text{-}1^+$ $CD4^+$ Tfh cell samples (GFP^+ and GFP^-), and $GFP^-CXCR5^-PD\text{-}1^-$ $CD4^+$ non-Tfh cells from PP cells of IL-21eGFP mice. Only DEG with $\log_2$ fold change (logFC) value ≥ 0.58 and p≤ 0.05 were included. (a) Tfh DEGs with similar expression levels in $GFP^+CXCR5^+PD\text{-}1^+$ and $GFP^-CXCR5^+PD\text{-}1^+$ $CD4^+$ cells. (b) Tfh DEG subset further downregulated in GFP^+Tfh than in to GFP^-Tfh cells. (c) Tfh DEG subset with higher upregulation in GFP^+Tfh than in GFP^-Tfh cells. Genes in the heatmaps are shown in Supplementary Tables 1-3. Complements Figure 1 of the manuscript.

Supplementary Figure S4

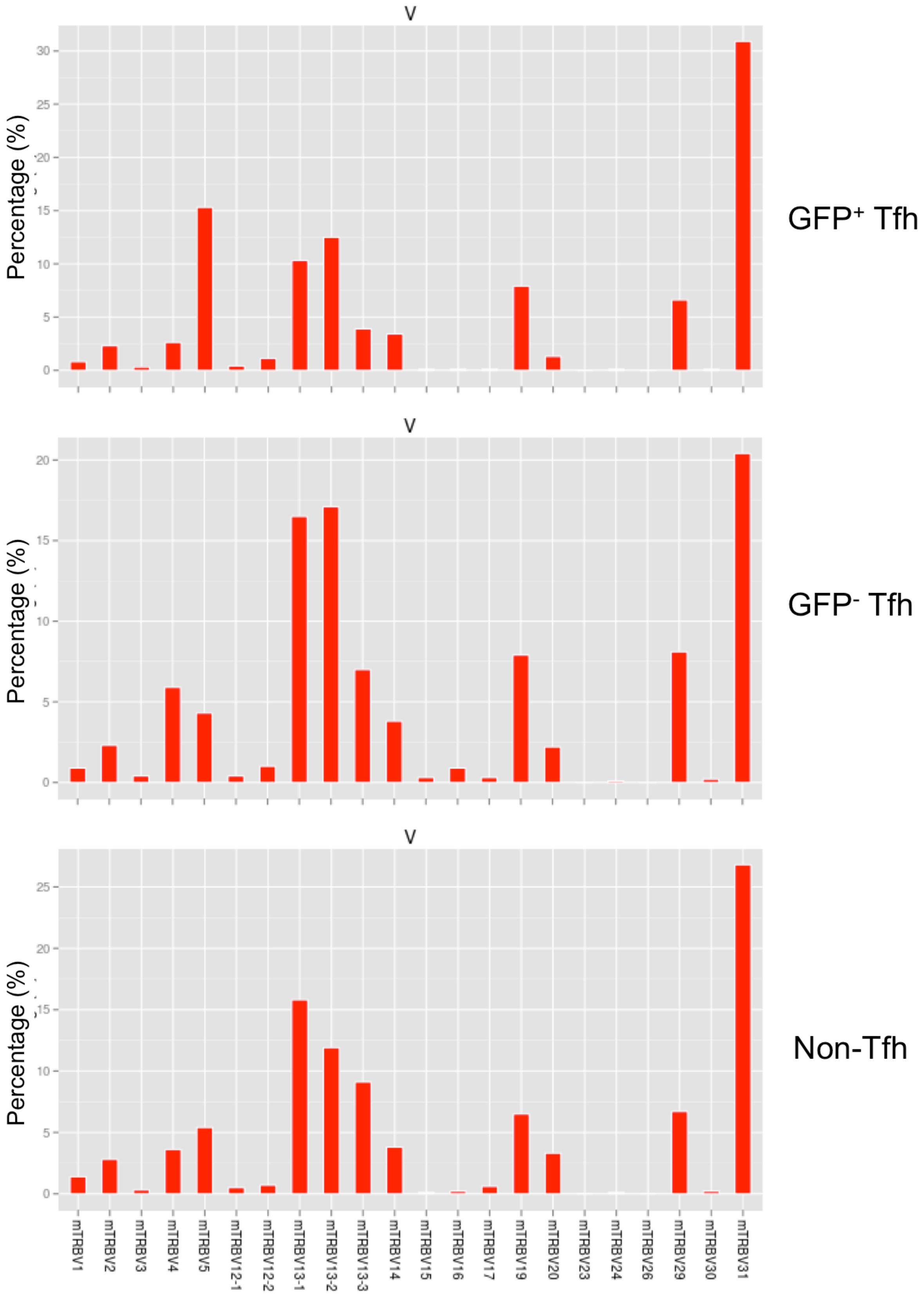

Supplementary Figure S4 GFP^{+}Tfh and GFP^{-}Tfh cells from PP have a diverse polyclonal TCRβ repertoire. Percentage of Vβ gene usage in sorted PP $CD4^{+}$ T cell populations from a representative IL-21eGFP mouse. Complements Figure 2 of the manuscript.

Supplementary Figure S5

a

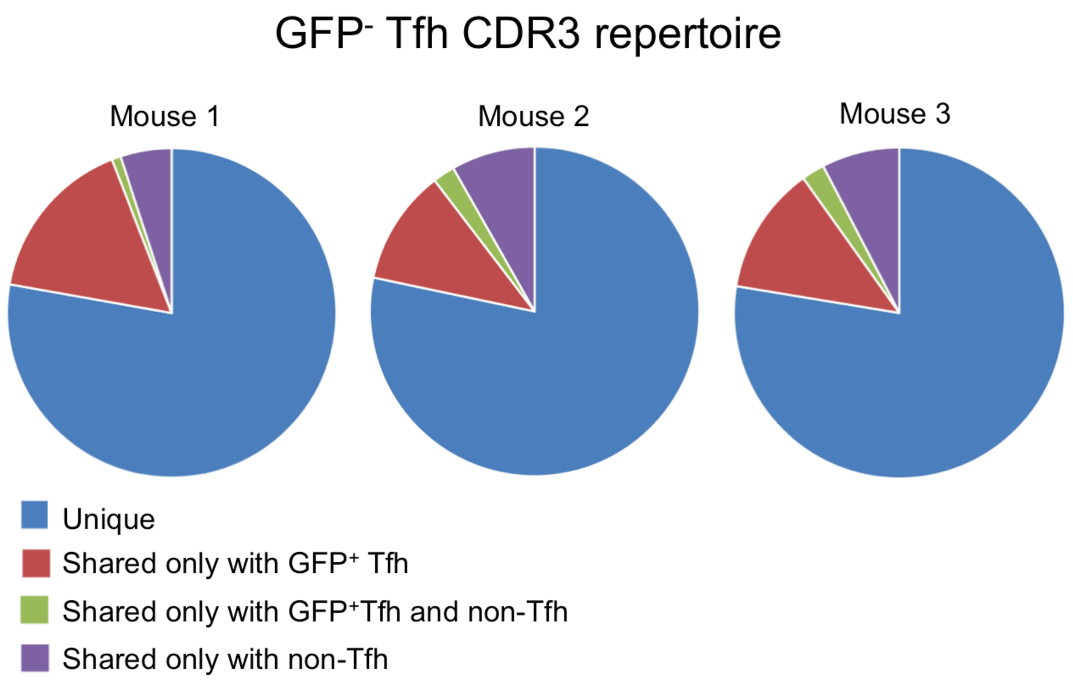

b

	GFP+Tfh M1	GFP-Tfh M1	Non-Tfh M1	GFP+Tfh M2	GFP-Tfh M2	Non-Tfh M2	GFP+Tfh M3	GFP-Tfh M3	Non-Tfh M3
GFP+Tfh M1	1								
GFP-Tfh M1	**0.181**	1							
Non-Tfh M1	0.012	0.028	1						
GFP+Tfh M2	0.017	0.017	0.004	1					
GFP-Tfh M2	0.024	0.031	0.010	**0.158**	1				
Non-Tfh M2	0.005	0.011	0.019	0.016	0.044	1			
GFP+Tfh M3	0.020	0.029	0.005	0.029	0.028	0.005	1		
GFP-Tfh M3	0.025	0.035	0.010	0.017	0.037	0.013	**0.172**	1	
Non-Tfh M3	0.006	0.010	0.017	0.003	0.009	0.021	0.015	0.036	1

Supplementary Figure S5 The TCRβ CDR3 repertoire of PP CD4^{+} T cell subsets.

(a) Overlap of the repertoire of unique TCR Vβ CDR3 sequences from GFP^{-}Tfh cells, with the repertoire of GFP^{+}Tfh and non-Tfh CD4^{+} cells in the same mouse. Each pie graphic represents all GFP^{-}Tfh cells' unique CDR3 sequences from one mouse (n=3). (b) Repertoire relatedness between paired CD4^{+} populations of individual mice as

determined by computational β diversity analysis. Complements Figure 2 of the manuscript.

Supplementary Figure S6

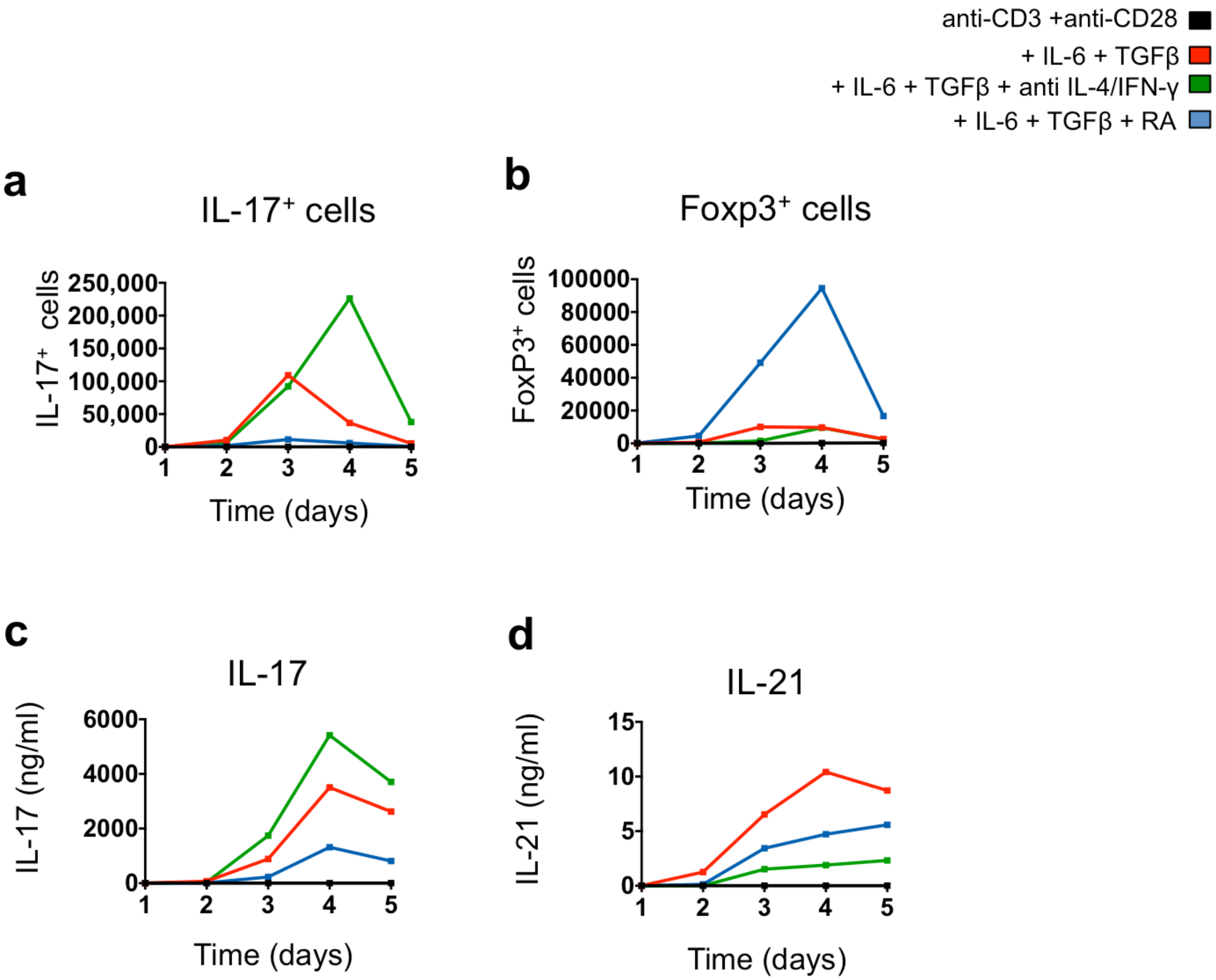

Supplementary Figure S6 Induction of IL-21 expression in $CD4^+$ T cells activated in the presence of TGFβ, IL-6 and RA. Splenocytes from naïve TBmc mice were stimulated with anti-CD3 and anti-CD28 with addition of indicated cytokines, blocking antibodies or RA. Number of (a) IL-17^+ and (b) $Foxp3^+$ $CD4^+$ cells as determined by intracellular staining. (c) IL-17 and (d) IL-21 levels in culture supernatants collected daily and measured by ELISA. Complements Figure 3 of the manuscript.

Supplementary Figure S7

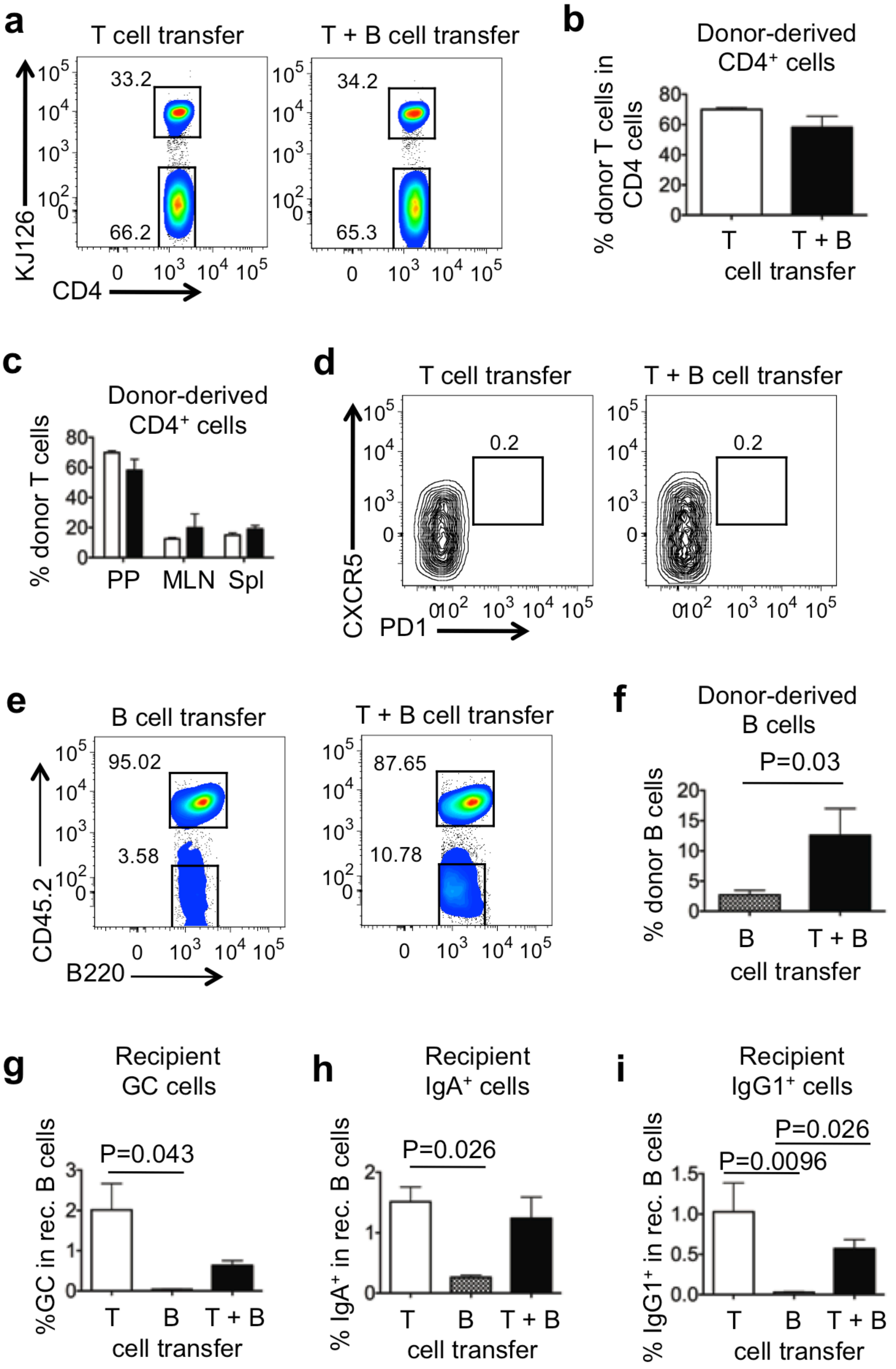

Supplementary Figure S7 Splenic GFP^-CD4^+ T cells IL-21eGFP mice traffic to PP where they differentiate into GFP^+Tfh cells and drive B cell activation. Splenic polyclonal GFP^-CD4^+ T cells from IL-21eGFP BALB/c mice were transferred alone (T) or with polyclonal B cells from $CD45.1^+$ BALB/c mice (T+B) into TBmc mice. Groups of TBmc mice also received $CD45.1^+$ B cells without T cells (B). 4 weeks post transfer, PP cells T and B cell populations in the recipient mice were analysed by flow cytometry. (a, b) Donor T cells, identified as $CD4^+KJ126^-$, expanded similarly in PP of recipient mice infused or not with polyclonal B cells. (a) Representative flow cytometry plots of gated $CD4^+$ T cells showing endogenous KJ1-26^+ CD4 T cell gate and donor KJ1-26^- CD4 T cell gate. (b) Percentage of donor $CD4^+$ cells in total PP $CD4^+$ cells. (c) Percentage of donor T cells in the $CD4^+$ T cell population of PP, mesenteric LN (MLN) and spleen (Spl) of recipient mice. (d) Representative flow cytometry plots of gated endogenous KJ1-26^+CD4^+ T cells in PP of recipient mice demonstrating absence of Tfh cells. (e-f) Larger expansion of donor $CD45.1^+$ B cells in PP of recipient mice co-transferred with polyclonal T cells. (e) Representative plots of gated $B220^+$ B cells showing endogenous $CD45.2^+$ B cell gate and donor $CD45.2^-$ B cell gate. (f) Percentage of donor $CD45.2^-$ $B220^+$ cells in total $B220^+$ cells from PP. (g-i) Differentiation of endogenous $CD45.2^+$ B cells into (g) $CD95^+GL7^+$ GC cells, (h) IgA^+ cells and (i) $IgG1^+$ cells. All bar graphs show mean + s.e.m of 6-8 mice per group. Statistical significance was determined by unpaired, two-tailed Mann Whitney U-test (f) or Kruskal-Wallis followed by Dunn's multiple comparisons test (g-i). P values are only shown for statistically significant differences ($p<0.05$). Complements Figure 4 of the manuscript.

Supplementary Figure S8

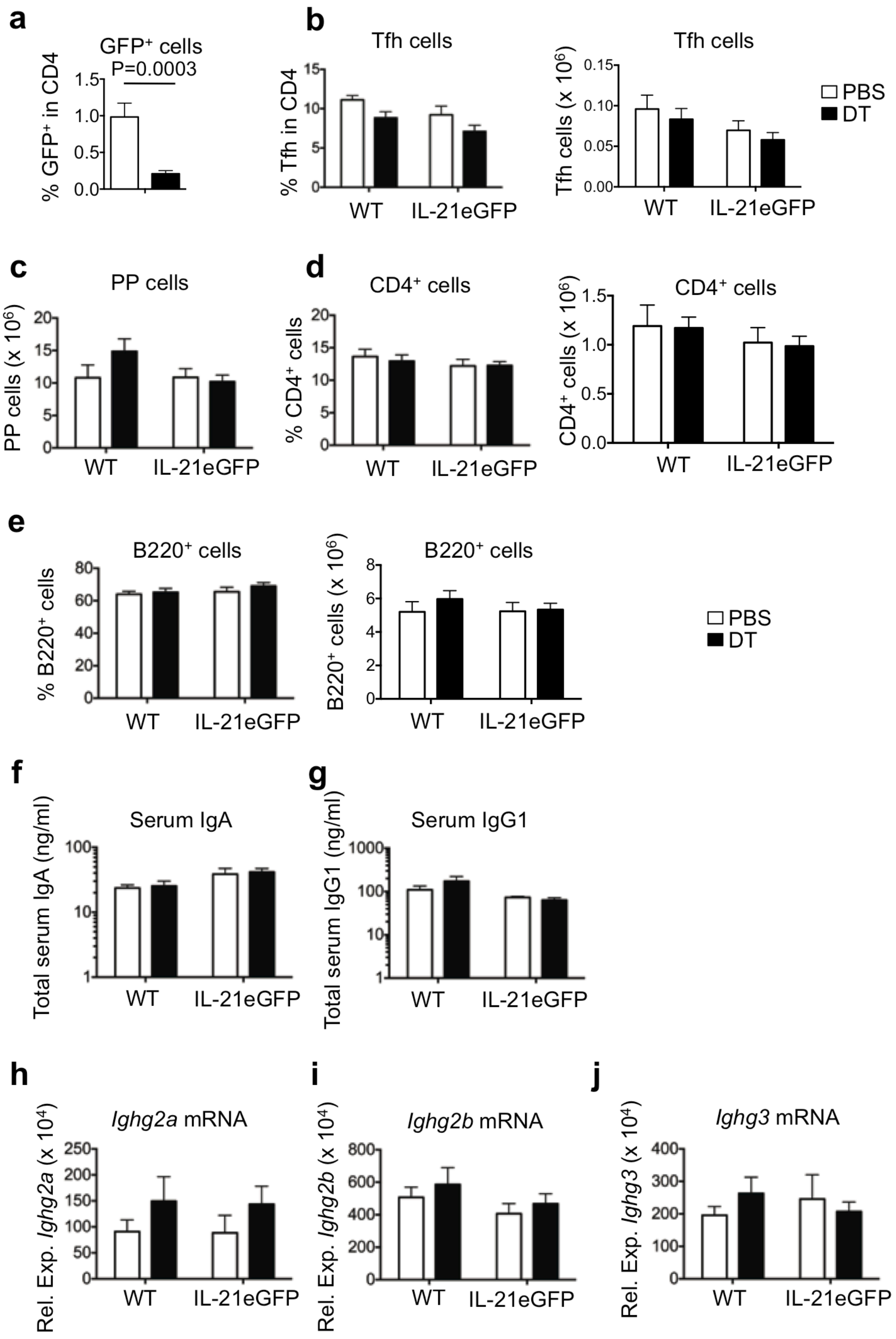

Supplementary Figure S8 DT depletion of GFP^+ cells in IL-21eGFP mice results in alterations of local B cell activation and antibody production in the PP. IL-21eGFP mice and non-transgenic WT littermates were treated with DT twice daily for 3 weeks from 3-6 weeks old. PP cells were analyzed at 6 weeks of age. (a) Total PP cells. (b) Percentage (left graph) and total numbers (right graph) of $CD4^+$ T cells in PP. (c) Percentage of GFP^+ cells in $CD4^+$ T cells in PP. (d) Percentage (left graph) and total numbers (right graph) of Tfh cells in $CD4^+$ T cells in PP. (e) Percentage (left graph) and total numbers (right graph) of $B220^+$ cells in PP. (f-g) Serum levels of (f) IgA and (g) IgG1 in 6 weeks old mice. (h-j) QPCR analysis of (h) *Ighg2a*, (i) *Ighg2b*, and (j) *Ighg3* transcripts in PP of 6 weeks old mice. Data are expressed as mean ± s.e.m. of 5-12 mice per group from two independent experiments. Statistical significance was determined by unpaired, two-tailed, Mann-Whitney U-test. P values are only shown for statistically significant differences ($p<0.05$). Complements Figure 7 of the manuscript.

Supplementary Figure S9

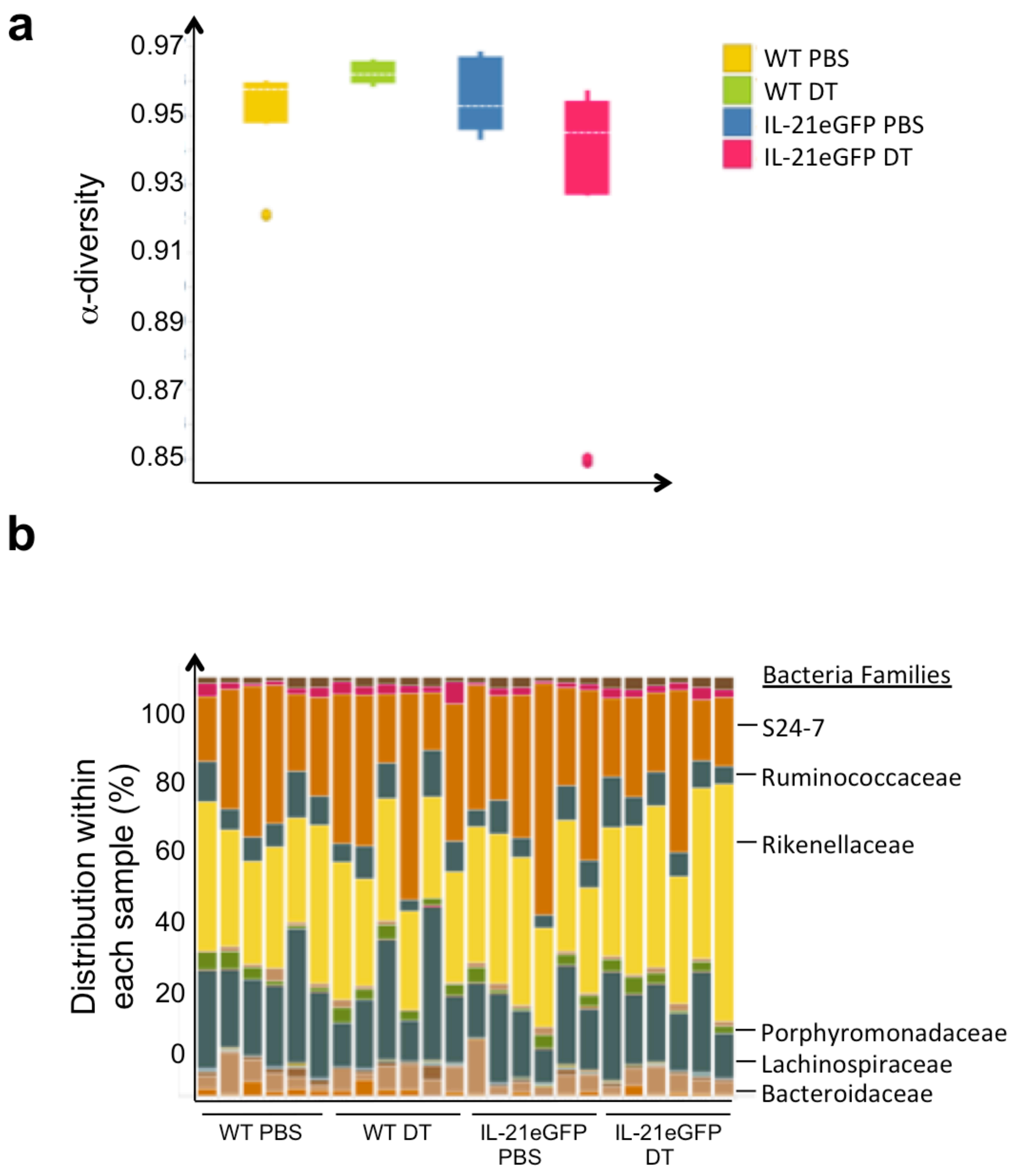

Supplementary Figure S9 Overall composition of gut microbiome was similar between WT and IL21eGFP mice. IL-21eGFP mice and non-transgenic WT littermates were treated with DT twice daily for 3 weeks from 3-6 weeks old. Genomic DNA was extracted from fecal samples and the 16S variable regions were amplified and sequenced. (a) α diversity analysis of the gut microbiome of WT and IL21eGFP mice treated with PBS or DT. No significant differences were observed. (b) Distribution of gut

microbe families within fecal samples from WT and IL21eGFP mice treated with PBS or DT. Complements Figure 7 of the manuscript.

SUPPLEMENTARY METHODS

- **Generation of IL-21eGFP mice**
- **Isolation of PP lymphocytes**
- **Antibiotic treatment**
- **Diphtheria toxin (DT) treatment**
- **16S RNA sequencing of fecal microbiome**
- **Flow cytometry and cell sorting**
- **RNA expression analysis by QPCR and microarray**
- **High throughput TCR sequencing**
- **ELISA**
- **Immunohistology**
- **List of antibodies used**
- **Primer sequences**
- **References**

Generation of IL-21eGFP mice. IL-21eGFP transgenic mice were generated using BAC technology. First, *Il21* upstream and downstream homologous sequences were ligated to the 5' and 3' ends respectively of a cassette encoding a DTR-eGFP fusion protein and the SV40 polyadenylation site in pGEM T vector [1]. The cassette was then recombined with the wild type *Il21* gene in BAC RP23-137F6 (Invitrogen-Life Technologies). The BAC insert was gel purified and injected into C57Bl/B6 blastocysts at the Transgenic Facility of the New York University School of Medicine (NYUSM). IL-21eGFP mice were genotyped by PCR using the primers TTATCCTCCAAGCCACAAGC and AAGTCGTGCTGCTTCATGTG. Of the 7 founders, one expressing GFP in $CD4^+$ T cells of Peyer's Patches (PP) was backcrossed to the BALB/c strain (The Jackson Laboratories) and to TBmc mice [2] for use in the study.

Isolation of Peyer's Patches lymphocytes. PP lymphocytes were prepared as previously described [3] with some modifications: PP were excised and epithelial cells removed by incubation in PBS with 1mM EDTA for two rounds of 10 minutes at 37°C and with 200 rpm rotation. Following washing with PBS, the remaining tissue was incubated with 50μg/ml Liberase™ (Roche) and 333μg/ml DNAse (Sigma) at 37°C during centrifugation at 100 rpm for 30 minutes, before a second 15 minute incubation with DNAse. A single cell suspension was generated by pressing the tissue through a cell strainer. Cells were then washed in RPMI-1640 supplemented with 10% fetal bovine serum (Invitrogen).

Antibiotic treatment. For broad ablation of the intestinal microbiota, a cocktail of antibiotics – ampicillin (0.5mg/ml; Sigma), metronidazole (0.5mg/ml; Acros Organic),

neomycin sulfate (1mg/ml; Fisher) and vancomycin hydrochloride (0.25mg/ml; Fisher) was supplied to mice in the drinking water. For ablation of gram negative bacteria a cocktail of metronidazole (0.5mg/ml), neomycin sulfate (1mg/ml) and polymyxin B (0.2mg/ml) was used. For ablation of gram positive bacteria, mice were treated with vancomycin (0.25mg/ml). The artificial sweetener Splenda was added to the water at 4mg/ml to encourage drinking. Treatments commenced at 2 weeks of age when mice were still in the breeding cages and continued after weaning. Control cages were kept on water containing Splenda alone. After weaning, experimental cages contained same sex transgenic and non-transgenic BALB/c littermates undergoing the same treatment. Both males and females were included in these experiments.

Diphtheria toxin (DT) treatment. To deplete GFP^+ cells, IL-21eGFP mice were injected i.p. with DT (5ng/g body weight; Sigma) diluted in 100μl PBS twice daily for three weeks. Control mice received 100μl PBS under the same treatment regimen. Non-transgenic WT BALB/c littermates underwent identical treatment.

Flow cytometry and cell sorting. Single cell suspensions were incubated with 10μg/ml anti-CD16/32 for 10 minutes at 4°C prior to surface labeling with antibody cocktails in labeling buffer (2% FBS and 0.1% NaN_3 in PBS) for 40 minutes at 4°C. Labeling for CXCR5 was carried out at 37°C for 30 minutes prior to any other surface labeling. Where indicated, intra-nuclear detection of FoxP3 or Bcl6 was conducted using the FoxP3/Transcription Factor staining buffer kit (eBioscience). Intracellular staining of cytokines was performed using BD Biosciences Cytofix/Cytoperm™ kit. Cells were analyzed using a FACsCanto or LSR II 5-laser flow cytometer (BD Biosciences) or

sorted using an Influx or FACsAria II 4-laser cell sorter (BD Biosciences), with FlowJo software (Tree Star) for data analysis. Purified cells were used for QPCR or adoptive transfer experiments. Details of all antibodies used are in a table below.

RNA expression analysis by QPCR and microarray. For quantitative PCR analysis (QPCR) of gene expression, RNA was obtained from cells using TRIzol (Invitrogen) and cDNA synthesized with SuperScript II reverse transcriptase as per manufacturer's instructions. QPCR for selected gene transcripts was carried out using primers (as listed below) on a BioRad cFX96 RT-PCR instrument. Gene expression was normalized to β-Actin.

Microarray expression analysis of triplicate samples of sorted $GFP^{+}CXCR5^{+}PD1^{+}$ Tfh, $GFP^{-}CXCR5^{+}PD1^{+}$ Tfh, and GFP^{-} $CXCR5^{-}PD1^{-}$ non-Tfh $CD4^{+}$ T cells from PP was performed as previously described using Affymetrix MoGene 1.0 ST arrays [4]. A list of Tfh differentially expressed genes (DEG) was generated using $\log_2$ fold change (logFC) value ≥ 0.58 and p≤ 0.05 as selection criteria. Microarray datasets can be found in NCBI GEO GSE77899.

High throughput TCRβ sequencing. $CD4^{+}$ T cells from PP of IL-21eGFP mice were sorted into $CXCR5^{+}PD\text{-}1^{+}GFP^{+}$Tfh, $CXCR5^{+}PD\text{-}1^{+}GFP^{-}$Tfh and $CXCR5^{-}PD\text{-}1^{-}$ naive cell populations using a LSRII 4-laser cell sorter (BD Biosciences). Total RNA was isolated using ARCTURUS® PicoPure® RNA Isolation Kit (Life Technologies) and immune repertoire amplicon libraries were prepared using 2ng of RNA as described [5]. Briefly, reverse transcription and amplification were performed with Mouse TCR beta, Illumina MiSeq, V-C gene Primers (iRepertoire Inc., Huntsville, AL, USA) and Qiagen One-Step

RT-PCR kit (Qiagen). In the primary PCR reaction barcode sequences for identifying and demultiplexing individual samples were added to the template while the Illumina adapters were added in the secondary PCR reaction using Qiagen Multiplex PCR Kit. Equimolar concentrations of secondary PCR products were pooled, electrophoresed in 2% agarose gel, and amplicons in the 400-450 bp range were gel purified. qPCR was performed (Kapa Biosystems) on the amplicon library to ascertain the loading concentration. The library was sequenced using Illumina MiSeq to generate 250bp paired end reads at a sequencing depth of 1 million reads per sample. Raw sequencing reads were submitted to iRepertoire® for analysis. In brief, raw reads were demultiplexed and processed using a SMART (**S**equencing, **M**osaic, **A**mplification, **R**eference, Frequency **T**hreshold) filter to remove sequencing errors, chimeric sequences, PCR artifacts, mismatches with reference sequences and low frequency CDR3s. Identical reads were collapsed and assigned to V,D,J segments using Smith-Waterman algorithm [6] for local sequence alignment with germline reference from IMGT (www.imgt.org). Raw and processed reads were provided by iRepertoire® for further analysis.

α and β diversity indices were generated using the vegan package in R v2.15.2/Bioconductor [6,7]. The α diversity index was determined using Shannon's Index. The β diversity index was determined using the "z" method of the betadiver function from vegan, specifically (log(2)-log(2*a+b+c)+log(a+b+c)/log(2), where "a" represents the number of shared sequences, and b and c represent the unique sequences in the two samples. The datasets of the TCRβ sequence analysis were deposited in NCBI Bioproject, accession PRJNA311496.

16S RNA sequencing of the fecal microbiome. Genomic DNA (gDNA) was isolated from stool samples of mice using the QIAmp DNA stool kit (QIagen, Valencia, CA) following manufacturers instructions. For amplification of the 16S variable regions (V4 to V5), PCR was performed using 10ng of stool gDNA with Long Amp Taq polymerase (New England Biolabs, Ipswich, MA) according to manufacturer's instructions. The reaction mix for the first round of amplification contains a specific forward primer and reverse primer binding to V4 & V5 regions, respectively. The forward primer (U519F Mod –CAGCMGCCGCGGTAAYWC) was modified from Baker et al. [8], while the reverse primer MetGen R-BSCCCGYCAATTYMTKTRAGT was modified from Teske and Sorenson [9]. Secondary PCR was carried out to further enrich for variable region sequences using communal primers. During the secondary PCR reaction, barcodes for identifying and de-multiplexing individual samples, and illumina adapter sequences, were added to the template. PCR cycling parameters consisted of initial denaturation for 30s at 94°C, followed by 15 cycles of 15 s at 94°C, 30 s at 45°C, and 30 s at 65°C with a final extension for 10 min at 65°C for the primary PCR reaction. For secondary PCR, the cycling parameters were the same except that amplification was carried out for 25 cycles. Equimolar concentrations of secondary PCR products were pooled and electrophoresed using 2% agarose gel. Amplicons in the size range of 550 bp were gel purified using the Qiaquick Gel Extraction Kit (Qiagen). Concentrations of gel purified libraries were estimated using the DNA 1000 kit (Agilent Technologies, Santa Clara, CA). QPCR was performed using quantified amplicon libraries (Kapa Biosystems, Wilmington, MA) to ascertain the loading concentration. The library was sequenced using Illumina MiSeq to generate 250bp Single end reads at a sequencing depth of about half a million reads per sample. These reads were trimmed for primers in Accelrys

Pipeline Pilot, USA (version 9.2.0, www.accelrys.com) and quality (cutoff=20) in BBDuk [http://sourceforge.net/projects/bbmap/]. Trimmed reads were mapped to Silva 119 OTU database [10] with QIIME version 1.8.0 [11] OTU picking script pick_closed_reference_otus.py using uclust [12]. β diversities were calculated using Unifrac [13] distances with beta_diversity_through_plots.py script, and alpha_diversity.py scripts in QIIME and alpha-diversity simpson index was calculated and compared for all the samples using compare_alpha_diversity.py script in QIIME. The association between metadata and microbial abundance was determined in MaAsLin (http://huttenhower.org/galaxy) by pairwise comparisons of DT-treated and PBS-treated control mice within WT and IL-21eGFP groups. QIIME and TIBCO Spotfire, USA (version 5.5.0, http://spotfire.tibco.com/) were used to make visualisations. Datasets for the microbiome analysis are in NCBI BioProject, accession number PRJNA293925.

ELISA. Feces were collected and weighed before disruption in protease inhibitor (Complete Mini, EDTA free [14]) at 100mg feces/ml. Fecal supernatants were prepared by centrifuging at 10,000 x g for 10 minutes at 4°C. ELISA was carried out on fecal supernatants for determination of the concentration of IgA, and on serum samples for IgA and IgG1 as described[2]. Cell culture supernatants were tested for levels of IL-17 and IL-21. Typically, pairs of unlabeled and biotinylated antibodies were used for plate coating and detection of the analyte, respectively. Plates were incubated with streptavidin conjugated to Horse Radish Peroxidase (HRP) and a colorimetric substrate to measure optical density. Purified cytokines and antibody isotypes were used to obtain a standard curves. All antibodies used are listed below.

Immunohistology. Segments of intestine containing PP were frozen in OCT (Tissue-Tek) and sectioned at 8µM. Sections were fixed with 1:1 acetone/methanol, washed in PBS and then incubated for 1 hour at room temperature in blocking buffer (PBS with 1% BSA and 20µg/ml anti-mouse CD16/32). Sections were then incubated overnight at 4°C with primary antibodies, washed with PBS and counterstained with Hoescht for 15 minutes at room temperature. Fluorescent images were captured with an Olympus FV1000 confocal microscope and analyzed with Olympus FluoView software.

List of antibodies used

Antigen	Clone	Conjugate	Source
Bcl6	K112-91	Alexa Fluor 647	BD Biosciences
CD3e	145-2C11	Unlabelled	BD Biosciences
CD4	GK1.5	eFluor 450	eBioscience
CD4	GK1.5	eFluor 605NC	eBioscience
CD4	L3T4	PerCP	BD Biosciences
CD4	RM4-5	APC	eBioscience
CD8	53-6.7	PE	eBioscience
CD11b	M1/70	PE	eBioscience
CD19	eBio1D3 (1D3)	eFluor 450	eBioscience
CD19	eBio1D3 (1D3)	PeCy7	eBioscience
CD25	PC61.5	APC	eBioscience
CD25	PC61.5	PE	BD Biosciences
CD28	37.51	Unlabelled	BD Biosciences
CD45R (B220)	RA3-6B2	PerCP	BD Biosciences
CD45R (B220)	RA3-6B2	PE-Cy7	eBioscience
CD45R (B220)	RA3-6B2	APC-eFluor780	eBioscience
CD45.2	104	APC	eBioscience
CD49b	DX5	PE	eBioscience
CD95	Jo2	PE-Cy7	BD Biosciences
CD138	281-2	Brilliant Violet 421	BioLegend
CD138	281-2	APC	BD Biosciences
CD278 (ICOS)	7E.17G9	PE	eBioscience
CD279 (PD1)	J43	APC	BD Biosciences
CD279 (PD1)	RMP1-30	eFluor450	eBioscience
CD279 (PD1)	J43	PE	BD Biosciences
CD326 (EPCAM)	G8.8	APC	eBioscience

Antigen	Clone	Conjugate	Source
CD326 (EPCAM)	G8.8	PE	eBioscience
CXCR5	2GB	PE-Cy7	BD Biosciences
FoxP3	FJK-16s	Alexa Fluor 647	eBioscience
GFP	Polyclonal IgG	Alexa Fluor 488	Invitrogen
IFNγ	XMG1.2	Unlabelled	BioLegend
IgA	Polyclonal IgG	Unlabelled	Southern Biotech
IgA	Polyclonal IgG	PE	Southern Biotech
IgA	Polyclonal IgG	HRP	Southern Biotech
IgD	11-26	PE	eBioscience
IgG1 F(ab')2	Polyclonal	Unlabelled	Southern Biotech
IgG1 F(ab')2	Polyclonal	Biotin	Southern Biotech
IgG1	RMG1-1	APC	BioLegend
IgG1	M1-14D12	PE-Cy7	eBioscience
IgG1	M1-14D12	PerCP-eFluor 710	eBioscience
IL-4	11B11	Unlabelled	BioLegend
IL-17A	TC11-18H10.1	PE	BioLegend
IL-17	50101	Unlabelled	R&D Systems
IL-17	Polyclonal IgG	Biotin	R&D Systems
IL-21	Polyclonal IgG	Unlabelled	R&D Systems
IL-21	Polyclonal IgG	Biotin	R&D Systems
Ly76	TER-110	PE	eBioscience
MHCII	M5/114.15.2	APC	eBioscience
MHCII	M5/114.15.2	PE	eBioscience
T and B cell activation antigen	GL7	Alexa Fluor 647	BD Biosciences
T and B cell activation antigen	GL7	FITC	BD Biosciences
TCR DO11.10	KJ126	PE	BioLegend
TCR Vβ2	B20.6	PE	BD Biosciences
TCR Vβ3	KJ25	PE	BD Biosciences
TCR Vβ4	KT4	PE	BD Biosciences
TCR Vβ5.1/5.2	MR9-4	PE	BD Biosciences
TCR Vβ6	RR4-7	APC	eBioscience
TCR Vβ7	TR310	PE	BioLegend
TCR Vβ8	F23.1	PE	BD Biosciences
TCR Vβ8.1/8.2	MR5-2	PE	BioLegend Biosciences
TCR Vβ8.3	8C1	PE	BioLegend
TCR Vβ10	B21.5	PE	eBioscience
TCR Vβ11	RR3-15	PE	BD Biosciences
TCR Vβ12	MR11-1	PE	BioLegend

Primer sequences

Gene	Forward primer	Reverse primer
B-Actin	TGACAGGATGCAGAAGGAGA	GTACTTGCGCTCAGGAGGA
IL-21	CGCCTCCTGATTAGACTTC	GCCCCTTTACATCTTGTGGA
IgA	AGTGGCGCATCATTCAAGT	CTGGGGTGGGAAGGTGTT
IgG1	AGTCTGACCTCTACACTCTG	CTTACAACCACAATCCCTG
IgG2a	TGACCTCTACACCCTCAGCA	AGGACAGGGCTTGATTGTGG
IgG2b	CACCACGGTGGACAAAAAAC	ACAGGGGTTGATTGTTGAA
IgG3	GCCAGCAAGACTGAGTTGAT	GGGTACTGGGCTTGGGTATT

References

1 Lahl, K. et al. Selective depletion of Foxp3+ regulatory T cells induces a scurfy-like disease. J Exp Med 204, 57-63 (2007).

2 Curotto de Lafaille, M. A. et al. Hyper immunoglobulin E response in mice with monoclonal populations of B and T lymphocytes. J Exp Med 194, 1349-1359 (2001).

3 Sheridan, B. S. & Lefrancois, L. Isolation of mouse lymphocytes from small intestine tissues. Curr Protoc Immunol Chapter 3, Unit3 19 (2012).

4 He, J. S. et al. The distinctive germinal center phase of IgE+ B lymphocytes limits their contribution to the classical memory response. J Exp Med 210, 2755-2771(2013).

5 Wang, C. et al. High throughput sequencing reveals a complex pattern of dynamic interrelationships among human T cell subsets. Proc Natl Acad Sci U S A 107, 1518-1523 (2010).

6 Black, P. E. Smith-Waterman algorithm, in Dictionary of Algorithms and Data Structures [online]. Vreda Pieterse and Paul E. Black, eds. 2006.

7 Jari Oksanen, F. G. B., Roeland Kindt, Pierre Legendre,, Peter R. Minchin, R. B. O. H., Gavin L. Simpson, Peter Solymos, M. & Wagner, H. H. S. a. H. vegan: Community Ecology Package. R package version 2.0-10. http://CRAN.R-project.org/package=vegan (2013).

8 Baker, G. C., Smith, J. J. & Cowan, D. A. Review and re-analysis of domain-specific 16S primers. J Microbiol Methods 55, 541-555 (2003).

9 Teske, A. & Sorensen, K. B. Uncultured archaea in deep marine subsurface sediments: have we caught them all? ISME J 2, 3-18 (2008).

10 Quast, C. et al. The SILVA ribosomal RNA gene database project: improved data processing and web-based tools. Nucleic Acids Res 41, D590-596 (2013).

11 Caporaso, J. G. et al. QIIME allows analysis of high-throughput community sequencing data. Nat Methods 7, 335-336 (2010).

12 Edgar, R. C. Search and clustering orders of magnitude faster than BLAST. Bioinformatics 26, 2460-2461 (2010).

13 Lozupone, C. & Knight, R. UniFrac: a new phylogenetic method for comparing microbial communities. Appl Environ Microbiol 71, 8228-8235 (2005).

14 Tian, J. et al. Toll-like receptor 9-dependent activation by DNA-containing immune complexes is mediated by HMGB1 and RAGE. Nat Immunol 8, 487-496 (2007).
